# Cytological, molecular, cytogenetic, and physiological characterization of a novel immortalized human enteric glial cell line

**DOI:** 10.3389/fncel.2023.1170309

**Published:** 2023-04-20

**Authors:** Lisa Zanoletti, Aurora Valdata, Kristina Nehlsen, Pawan Faris, Claudio Casali, Rosalia Cacciatore, Ilaria Sbarsi, Francesca Carriero, Davide Arfini, Lies van Baarle, Veronica De Simone, Giulia Barbieri, Elena Raimondi, Tobias May, Francesco Moccia, Mauro Bozzola, Gianluca Matteoli, Sergio Comincini, Federico Manai

**Affiliations:** ^1^Department of Biology and Biotechnology “L. Spallanzani”, University of Pavia, Pavia, Italy; ^2^Department of Chronic Diseases and Metabolism (CHROMETA), KU Leuven, Leuven, Belgium; ^3^InSCREENeX GmbH, Braunschweig, Germany; ^4^Department of Biology, College of Science, Salahaddin University-Erbil, Erbil, Iraq; ^5^Immunohematology and Transfusion Service, I.R.C.C.S. Policlinico San Matteo, Pavia, Italy; ^6^University of Pavia, Pavia, Italy

**Keywords:** enteric glial cells, enteric nervous system, transgene immortalization, viral transduction, immortalized human cell line

## Abstract

Enteric glial cells (EGCs), the major components of the enteric nervous system (ENS), are implicated in the maintenance of gut homeostasis, thereby leading to severe pathological conditions when impaired. However, due to technical difficulties associated with EGCs isolation and cell culture maintenance that results in a lack of valuable *in vitro* models, their roles in physiological and pathological contexts have been poorly investigated so far. To this aim, we developed for the first time, a human immortalized EGC line (referred as ClK clone) through a validated lentiviral transgene protocol. As a result, ClK phenotypic glial features were confirmed by morphological and molecular evaluations, also providing the consensus karyotype and finely mapping the chromosomal rearrangements as well as HLA-related genotypes. Lastly, we investigated the ATP- and acetylcholine, serotonin and glutamate neurotransmitters mediated intracellular Ca^2+^ signaling activation and the response of EGCs markers (*GFAP*, *SOX10*, *S100β*, *PLP1*, and *CCL2*) upon inflammatory stimuli, further confirming the glial nature of the analyzed cells. Overall, this contribution provided a novel potential *in vitro* tool to finely characterize the EGCs behavior under physiological and pathological conditions in humans.

## 1. Introduction

The Enteric Nervous System (ENS), known as “the second brain” in the gut, regulates gastrointestinal (GI) physiology, by controlling intestinal motility, secretion, blood circulation, and inflammatory response (Rühl et al., 2004; Bassotti et al., 2007). Although for many years enteric glial cells (EGCs), the largest component of the ENS, have been ascribed the unique role as supportive to neuronal functions by supplying nutrients to the enteric neurons, in the last decades, they have been known to be involved in several biological processes such as the regulation of intestinal homeostasis, maintenance of the epithelial barrier integrity, and gut defense (Seguella and Gulbransen, 2021). EGCs were originally identified in the last years of the XIX century, while detailed morphological analyses and identification of specific markers were performed secondly (Gabella, 1972; Jessen and Mirsky, 1980; Gabella, 1981). These astrocyte-like cells are primarily present in the submucosal and myenteric plexuses with ratios of 1.3 and 5.9 compared to neurons, respectively (Hoff et al., 2008). Based on their location in the gut, four types of EGCs can be distinguished. Myenteric glia and submucosal glia (Type I) are associated with neuronal cell bodies in the myenteric and submucosal plexus, while type II EGCs are located within the nerve fiber bundles connecting myenteric glia. Type III cells are in the mucosa and elongated type IV cells in the longitudinal muscle layer (Hanani and Reichenbach, 1994; Gulbransen and Christofi, 2018; Seguella and Gulbransen, 2021). The main markers for EGCs identification are GFAP, S100β, and SOXE (Sox8/9/10). GFAP is the primary protein involved in the formation of intermediate filaments (IF) in astrocytes. This protein is expressed both in Central (CNS) and Peripheral Nervous Systems (PNS) in different isoforms and splice variants, thus contributing to different biological processes, such as injury healing, gliosis, and inflammation (Middeldorp and Hol, 2011; Yang and Wang, 2015). S100β is a cytoplasmic EF-hand type Ca^2+^-Zn^2+^ binding protein of the S100 family, which plays a key role in maintaining microenvironmental homeostasis as well as in inflammatory responses in a concentration-dependent manner. Particularly, over-expression and secretion of S100β lead to the NF-kB-mediated production of iNOS and nitric oxide (NO) by binding to Receptor for Advanced Glycation Endproducts (RAGE), with the consequent activation of MyD88 (Rühl, 2005; Cirillo et al., 2011a). Finally, SOXE is a transcription factor group expressed in the mature EGCs, mainly adopted for quantitative analyses (Hoff et al., 2008; De Giorgio et al., 2012).

Recent studies demonstrated the involvement of enteric glia in inflammation and immune response by regulating neuroendocrine signaling and antigen-presenting mechanisms (Gulbransen and Christofi, 2018). Moreover, EGCs seem to be involved in the pathogenesis of different intestinal disorders, such as inflammatory bowels diseases (IBDs) and celiac disease, and recently they have been identified as part of the tumor microenvironment in colorectal cancer, pointing to these cells as new potential therapeutic targets (Savidge et al., 2007; Cirillo et al., 2009; von Boyen et al., 2011; Li et al., 2018; Pochard et al., 2018; Valès et al., 2019). However, due to technical difficulties, *in vitro*, *ex vivo,* and *in vivo* enteric glia studies are limited (Rühl et al., 2001a; Middlemiss et al., 2002; Grundmann et al., 2015; Rosenbaum et al., 2016; Le Berre-Scoul et al., 2017; D'Errico et al., 2018; Wang et al., 2018; Cerantola et al., 2020). Indeed, as reported by Soret et al. (2013), although for *in vitro* experiments EGCs can be isolated with different methods from guinea pig-, mouse-, rat-, and human- specimens, these methodologies require several passages that can affect the overall quality. Hence, possible cross-contamination with other cell types and EGCs de-differentiation represent a critical point in the study of their role in health and disease, thereby leading to the development of new standardized EGC models.

In this work, by making use of an established lentiviral transduction protocol (Lipps et al., 2018), we generated a novel immortalized human EGC line derived from the myenteric plexus (MP) which may represent a valuable tool to bridge the gap in *in vitro* EGCs knowledge.

## 2. Materials and methods

### 2.1. Human and murine EGCs isolation and established human cell lines culture conditions

Primary human EGCs (hEGCs) were isolated from the colon of a 75-year-old female donor. The tissue was obtained from Tissue Solutions (Glasgow, Scotland, United Kingdom) under ethics approval and donor consent (Declaration of Helsinki 1964 and its later amendments). Tissue (roughly 2 cm^3^) was collected and stored in HypoThermosol medium (BioLife Solutions, Bothell, WA, United States). The myenteric plexus (MP) was then isolated as described (Soret et al., 2013), cut into 1–2 mm fragments, and incubated for 20 min at 37°C. The fragments were homogenized with repeated pipetting in pre-warmed Digest Medium (6 mL/g of bioptic material) composed of Liver Digest Medium (Thermo Fisher Scientific, Waltham, MA, United States) supplemented with 2000 U of DNAse I, 10 mg of Liberase (Sigma-Aldrich, Burlington, MA, United States), 1 mL of trypsin 2.5% and 30 μL (stock solution 100 U/mL) of collagenase type I (Thermo Fisher Scientific). After incubation, the cell/tissue suspension was passed through a nylon mesh (100 μm) and centrifuged twice for 5 minutes at 200 x g. Cells were then cultivated with appropriate hECGs medium in plates pre-coated with hAEC Coating solution (InSCREENeX, Braunschweig, Germany) at 37°C and 5% CO_2_.

Murine primary EGCs (mEGCs) were isolated as described (Ibiza et al., 2016). Briefly, the muscularis layer and the submucosa were separated using a dissection microscope. The lamina propria was then scraped mechanically from the underlying mucosa using a coverslip. The isolated tissue was subsequently digested with Liberase and DNase I (Sigma-Aldrich) in RPMI (Euroclone, Milan, Italy) supplemented with 1% HEPES, 1% sodium pyruvate, 1% L-glutamine, 0.1 mg/mL streptomycin, 100 units/mL penicillin, and 0.1% of β-mercaptoethanol (Sigma-Aldrich) for 40 min at 37°C. The cell suspension was then passed using a 100 μm filter.

Human established low-grade astrocytoma Res186 (Bobola et al., 2005), high-grade astrocytoma T98G cells, and BJ hTERT human fibroblasts (ATCC, Guernsey, Ireland), used as controls, were cultivated in D-MEM medium supplemented with 10% FBS, 100 units/mL penicillin, 0.1 mg/mL streptomycin and 1% L-glutamine (Euroclone), at 37°C and 5% CO_2_ atmosphere.

### 2.2. hECGs lentiviral transduction and identification of integrated genes

hEGCs were immortalized as described (Lipps et al., 2018). In detail, hEGCs were transduced with self-inactivating lentiviral vectors after they reached 80% of confluence through different rounds of lentiviral infection with a MOI between 1 and 5. The lentiviral transduced genes used are listed in Supplementary Table S1. Cells were incubated overnight with lentiviral vectors at 37°C/5% CO_2_ in culture medium supplemented with Polybrene (8 μg/mL). After medium removal, 30 independent clones were selected for growing features. Among these, a relevant clone, hereafter referred to as ClK, was selected for further characterization and the cumulative population doubling level (cPDL) was calculated, according to ATCC’s recommendations. The integrated lentiviral genes were then confirmed through a PCR scheme, using a consensus forward primer (GGAGGCCTAGGCTTTTGCAA) located within the SV40 promoter sequence coupled with genes-specific reverse primers (Supplementary Table S1). Genomic DNA of the transduced cells was extracted with DNAzol reagent (Gibco Fisher Scientific, Dublin, Ireland), according to the manufacturer’s instructions. PCR was performed on 0.5 μg of template using the Mango-Taq Polymerase Kit (PJK-Biotech, Kleinblittersdorf, Germany). The adopted amplification protocol consisted of 40 cycles: 94°C (30 s)/55°C (45 s)/72°C (45 s). The presence/absence of the investigated genes was evaluated by agarose gel (2% *w/v*) electrophoresis.

### 2.3. Molecular characterization of ClK clone

Cumulative population doubling level (cPDL) was calculated following the ATCC guidelines, specifically *n* = 3.32 (logUCY-logl) + X, with n indicating the final PDL number at a given subculture, UCY representing the cell yield at that point, l indicating the cell number used as inoculum to start the subculture, and X representing the doubling level of the inoculum used to initiate the subculture. The expression of the enteric glial-specific markers (i.e., GFAP, SOX10, and S100β) and HLA class II molecules was analyzed using immunofluorescence. To this purpose, ClK cells (2×10^4^) were seeded on coverslips pre-coated for 2 h at 37°C/5% CO_2_ with huAEC Coating Solution (InSCREENeX) and cultured for 24 h. The fixation step was performed after a wash with PBS using 4% paraformaldehyde (PFA) for 15 min at room temperature (RT). Again, the coverslips were washed three times with PBS and incubated with 0.1% saponin (*v/v*) in PBS for 15 min (RT). Cells were then incubated for 1 h at RT with primary antibodies anti-GFAP, anti-SOX10, anti-S100β (Immunological Sciences, Rome, Italy), and anti-HLA-DQA1 (Abcam, Cambridge, United Kingdom) diluted 1:30 in 5% non-fat milk in PBS (*w/v*). After three washes with PBS, species-specific AlexaFluor633- and AlexaFluor488-labeled secondary antibodies (Thermo Fisher Scientific) were used at final dilution of 1:30 for 1 h (RT). Finally, after three washes with PBS, slides were stained with DAPI (0.4 μg/mL) (Sigma-Aldrich) for 8 min (RT). After a wash with distilled water, slides were mounted adding a drop of Dako Fluorescence Mounting Medium (DAKO, Jena, Germany) and sealed. Fluorescence signals were visualized using LEICA TCS SP8 STED 3X confocal microscope (Leica, Wetzlar, Germany). Specificity of the signal was assessed through negative controls, e.g., anti-Naspin antibody (Immunological Sciences).

The expression of CD31, CD45, CD271, and CD326 proteins was analyzed by flow cytometry. ClK cells (5×10^4^) were seeded in a pre-coated 6-multiwell plate until they reached 100% of confluence (48 h post-seeding). Then, cells were detached and centrifuged at 500 x g for 3 min at 4°C. After the removal of the supernatant, cells were stained with L/D efluor (Thermo Fisher Scientific) diluted 1:400 in PBS for 30 min at 4°C in the dark. Cells were then centrifuged as described and Fc block (1:100 in FACS buffer) was performed for 15 min at 4°C. After this step, cells were centrifuged at 500 x g for 3 min and incubated with extracellular staining mix containing specific anti-CD31-PE (Diluition: 1:300), CD45-PE-Cy5 (Diluition: 1:300), CD271-PE and CD326-PE-Cy7 (Diluition: 1:100) primary antibodies (BioLegend, San Diego, CA, United States) for 20 min at 4°C in the dark. Finally, cells were centrifuged as described, washed in appropriate buffer, and then resuspended into 200 μL of FACS buffer. The analysis was performed by BD FACSymphony A5 flow cytometer (BD, Franklin Lakes, NJ, United States) setting Forward Scatter (FSC) > 200 to exclude cellular debris.

### 2.4. Real-time PCR expression analysis

Total RNA was extracted from ClK cells with RNeasy Plus Micro Kit (QIAGEN, Hilden, Germany) according to manufacturer’s instructions and RNA quantification was performed using Nanodrop 1,000 (Thermo Fisher Scientific, Waltham, MA, United States). *GFAP*, *SOX10*, *S100β*, *PLP1*, and *GAPDH* cDNAs were obtained using random hexamers primers (Applied Biosystems, Forster City, CA, United States) as reported (Comincini et al., 2013). The genes, whose forward and reverse primers are reported in Supplementary Table S2, were amplified with LightCycler 480 SYBR Green I Master (Roche, Basel, Switzerland). Real-time PCR was performed using 2 μg of each cDNA amplified by means of Step-One PCR instrument (Applied Biosystems), with the following thermal profile: incubation of 95°C for 300 s, followed by 45 cycles of denaturation at 95°C for 10 s, annealing at 60°C for 15 s and elongation at 72°C for 15 s with fluorescence collection. After incubation at 95°C for 5 s, melting curve analysis was performed from 60 to 95°C, collecting data every centigrade degree (5 readings/°C). Samples were analyzed in duplicate, data were normalized to *GAPDH* and relative quantification (Schmittgen and Livak, 2008) was employed to calculate relative changes in gene expression.

### 2.5. HLA genotyping

Genomic DNA was isolated from ClK cells (1×10^6^) using Maxwell CSC Blood DNA automated Purification System (Promega, Madison, WI, United States) following the manufacturer’s instructions. HLA class I and II genotyping were performed with sequence-specific oligonucleotide-primed polymerase chain reaction (PCR-SSO) using the LABScan3D system (One Lambda Inc., Canoga Park, CA, https://www.graphpad.com/scientific-software/prism/) based on the Luminex xMAP technology (Luminex, Austin, Texas, United States). This technology was applied with LABType commercial kits (One Lambda Inc.) for class I HLA-A, B, C, and class II HLA-DRB1, DQA1, DQB1 genotyping, CWD (Common and Well-Documented alleles) and XR (High Resolution). The adopted amplification protocol occurred in 5 cycles (96°C x 20 s/70°C x 20 s/62°C x 20 s) with additional 30 cycles (96°C x 10 s/70°C x 15 s/62°C x 20 s). Amplifications were then evaluated by 2% agarose pre-casting electrophoresis.

### 2.6. Metaphase spread preparation

ClK cells (6×10^5^ at passage 21, p21) were seeded in 10 cm diameter Petri dishes. After 24 h, to accumulate metaphase-blocked cells, nocodazole (Sigma-Aldrich) was added to the cultures at a final concentration of 1.34 μM for 2 h at 37°C and 5% CO_2_. Subsequently, cells were detached using 0.5 ml of trypsin–EDTA (Thermo FisherScientific) and resuspended in PBS. Samples were centrifuged at 2200 x g for 10 min and resuspended in 10 ml of pre-warmed hypotonic solution (75 mM KCl) for 15 min at 37°C. Then, after another centrifugation at 2200 x g for 10 min, the pellet was resuspended in 10 ml of fixative solution (methanol and acetic acid, 3:1) for 45 min at −20°C. This passage was repeated once using fresh fixative solution. Cells were then centrifuged as before and resuspended in an appropriate volume of fresh fixative solution, according to the pellet size. Finally, the cell suspension was dropped on microscope slides pre-treated with fixative solution, and air-dried. Slides were then stained with DAPI (0.4 μg/mL) (Sigma-Aldrich, Burlington, MA, United States) for 8 min (RT). After a wash with distilled water, slides were mounted adding a drop of Dako Fluorescence Mounting Medium (DAKO) and sealed. Visualization was performed using a fluorescence microscope Axioplan (Zeiss, Oberkochen, Germany) provided with a Charged-Couple Device (CCD) camera (Photometrics). A sample of 100 images was collected and analyzed.

### 2.7. BAC extraction

Specific human BAC probes, with pBACe3.6 backbone, were purchased from BACPAC Human Resources (Emeryville, CA, United States). The probes used for Fluorescence *in Situ* Hybridization (FISH) experiments were RP11-160F8 (5q11.2, coordinates: 54.033.661–54.188.673) and RP11-69A18 (5q35.1, coordinates: 171.790.208–171.957.002). The purified *E. coli* LB stabs were propagated on LB agar with chloramphenicol (12.5 μg/mL) and single colonies were then isolated and expanded overnight in LB with antibiotic at 37°C in agitation. The obtained cultures were collected and centrifuged at 9600 x g for 30 min. After supernatant removal, the pellet was resuspended in 5 mL of P1 buffer (Tris–HCl 50 mM, pH 8; EDTA 10 mM; RNasi A 100 μg/mL) before the addition of 10 mL of P2 buffer (NaOH 200 mM; SDS 1%). The tubes were incubated for 5 min after gentle mixing. Later, 10 mL of P3 buffer (K-acetate 3 M) were added and the subsequent incubation was performed for 15 min at 4°C. The tubes were centrifuged at 9600 x g for 1 h and the supernatant was collected and centrifuged again for 45 min at 9600 x g. The obtained sample was loaded and purified using a QIAGEN-tip 100 column from the Qiagen Plasmid Mini Kit (QIAGEN, Hilden, Germany), according to manufacturer’s instructions. The final elution was centrifuged at 9600 x g for 45 min after the addition of 3.5 mL of isopropanol. The pellet was then resuspended in 70% ethanol and centrifuged again at 9600 x g for 20 min. After air-drying, the pellet was finally resuspended in a proper volume of sterile water. The purified BACs were digested using *EcoRI* enzyme and loaded onto 1% agarose gel to verify the presence of the inserts. BAC DNA quantification was performed using NanoDrop 1,000 spectrophotometer (Thermo Fisher Scientific).

### 2.8. Fluorescence *in situ* hybridization

For probes labeling, i 4 μL of Biotin/Digoxigenin-Nick Translation Mix (Roche), 1 μg of BAC probes, and sterile water were added to reach the final volume of 20 μL. The microtubes were then incubated at 15°C for 3 h. The reaction of nick translation was blocked using 1 μL of EDTA (25 mM, pH 8) before the addition of 10 μL of salmon sperm DNA (1 μg/μL), 2 μL of dextran blue (stock solution 1.8%, filtered), 10 μL of Roche human COT DNA (1 μg/μL), 17.2 μL of ammonium acetate (2.14 M, pH 8) and 151 μL of pre-refrigerated 100% ethanol. The samples were incubated for 2 h at −20°C and then centrifuged at 1100 x g for 20 min (RT). After supernatant removal, the pellets were dried, resuspended in 35 μL of hybridization solution (25% formamide, 10% dextran sulfate, 1% tween-20, 2X SSC), and stored at −20°C. Next, slides were incubated overnight at 37°C and then denatured for 2.5 min at 78°C after the addition of 150 μL of 70% formammide (Sigma-Aldrich), 2X saline sodium citrate (SSC, Sigma-Aldrich), and sterile water. After the denaturation step, slides were treated with 2X SSC (4°C) for 2.5 min and then dehydrated through the ethanol series (75–95-100%), 3 min each. Probes were denatured at 80°C for 8 min and then stored in ice to block the reaction.

For *in situ* hybridization and probe detection, on each slide, 15 μL of the labeled probe were added. The slides were then stored overnight at 37°C in a moisture chamber. Post-hybridization washes (3 washes of 5 min each) were performed using formamide 50% 2X SSC at 45°C, followed by 3 washes of 5 min with 2X SSC at 45°C. Slides were immediately permeabilized at 37°C for 30 min by adding 60 μL of 3% BSA in 0.1% Tween-20 4X SSC. Probes were incubated at 37°C for 30 min with anti-DIG and anti-BIO antibodies (Abcam), respectively conjugated to rhodamine and FITC fluorophores and diluted in 1% BSA, 0.1% Tween20 and 4X SSC. Three washes of 5 min each in 4X SSC and 0.1% Tween20 were then performed at 42°C. Slides were then treated with 60 μL of species-specific secondary antibodies labeled with rhodamine and FITC (1%BSA, 0.1% Tween20, 4X SSC) and further incubated at 37°C for 30 min. Two washes were performed as previously described, followed by a third one in 4X SSC (RT). Slides were covered with DAPI (0.4 μg/mL) (Sigma-Aldrich) and incubated for 8 min (RT). After a wash with distilled water, slides were further mounted by adding a drop of Dako Fluorescence Mounting Medium (DAKO) and sealed. Finally, probe detection was performed using an Axioplan fluorescent microscope (Carl Zeiss) provided with a Charged-Couple Device (CCD) camera (Photometrics).

### 2.9. Electron microscopy analysis

ClK ultrastructure analysis was performed by transmission electron microscopy (TEM). Cells (1×10^6^, p21) were centrifuged at 800 x g for 5 min and fixed with 2.5% glutaraldehyde in PBS for 2 h (RT). Cells were then washed and rinsed in PBS (pH 7.2) overnight and post-fixed in 1% aqueous OsO_4_ (Sigma-Aldrich) for 1 h (RT). Cells were pre-embedded in 2% agarose in water, dehydrated in acetone, and finally embedded in epoxy resin (Electron Microscopy Sciences, EM-bed812). Ultrathin sections (60–80 nm) were collected on nickel grids and stained with uranyl acetate and lead citrate. The specimens were observed with a JEM 1200 EX II (JEOL, Peabody, MA, United States) electron microscope, equipped with the MegaView G2 CCD camera (Olympus OSIS, Tokyo, Japan) and operating at 120 kV. The morphology of organelles (at least 20 for each type) was then analysed by two independent evaluators.

### 2.10. Flow cytometry analysis

For ClK cells (p21), mitochondrial membrane potentials, intracellular reactive oxygen species (ROS), and cell proliferation indexes were determined through flow cytometry analyses using Muse Cell Analyzer and dedicated kits (MitoPotential, Oxidative Stress and Ki-67 Assays Luminex), as described (Carriero et al., 2021; Slivinschi et al., 2022).

For MitoPotential aanalysis, after trypsinization and collection, cells were washed in Assay Buffer 1X, and resuspended in 100 μL of the same solution. Then, 95 μL of pre-diluted MitoPotential Reagent (1:1000) were added and cells were subsequently incubated for 25 min at 37°C. Finally, 5 μL of 7-AAD were added and the samples were analyzed after an incubation of 5 min in the dark (RT).

For oxidative stress analysis, after trypsinization and collection, cells were washed in Assay Buffer 1X and the pellet was resuspended in 10 μL of Assay Buffer 1X. Then, 190 μL of pre-diluted Oxidative Stress Reagent (1:800) were added and cells were subsequently incubated for 30 min at 37°C before the analysis. For Ki-67 proliferation analysis, after trypsinization and collection, cells were washed with PBS and resuspended in 1X Fixation solution for 15 min (RT). Then, cells were treated with Permeabilization solution for 15 min (RT) and subsequently incubated with Assay Buffer 1X for the same time interval. Subsequently, Muse Hu IgG1-PE (isotypic control) or Hu Ki67-PE antibodies were added, mixed, and incubated for 30 min (RT) before the analysis.

### 2.11. Ca^2+^ signals measurements

Ca^2+^ imaging was carried out by bathing EGCs in Physiological Salt Solution (PSS, 150 mM NaCl, 6 mM KCl, 1.5 mM CaCl2, 1 mM MgCl_2_, 10 mM glucose, 10 mM HEPES). In Ca^2+^-free solutions, Ca^2+^ was replaced with 2 mM NaCl with the addition of 0.5 mM EGTA. Solutions were titrated to pH 7.4 with NaOH. The osmolarity of PSS was measured with an osmometer (WESCOR 5500, Logan, UT, United States) and was equal to 338 mmol/Kg.

Ca^2+^ imaging was performed as described (Faris et al., 2020; Astesana et al., 2021). Briefly, cells (2 × 10^4^) were plated on round glass (8 mm) coverslips coated with huAEC Coating Solution (In-SCREENeX). The next day cells were loaded with fura-2 acetoxymethyl ester at the final concentration of 4 μM (Fura-2/AM, 1 mM stock in DMSO) in PSS for 30 min at 37°C and 5% CO2. After de-esterification in PSS for 15 min, the coverslip was mounted in a small size Petri dish, and cells were observed under an upright epifluorescence Axiolab microscope (Carl Zeiss) equipped with a Zeiss 40X Achroplan objective (water-immersion, 2.0 mm working distance, 0.9 numerical aperture). Cells were alternatively excited at 340 and 380 nm by using a filter wheel (Lambda 10, Sutter Instrument, Novato, CA, United States). Fluorescent emission was detected at 510 nm using an Extended-ISIS CCD camera (Photonic Science, Millham, United Kingdom). The fluorescent signals were measured and plotted on-line from 10 up to 40 selected regions of interest (ROIs), each corresponding to a well-defined single cell. The intracellular Ca^2+^ concentration was monitored by measuring for each ROI the ratio of the mean fluorescence emitted at 510 nm when exciting alternatively at 340 and 380 nm (Ratio F340/F380). An increase in [Ca^2+^]i causes an increase in the ratio (Faris et al., 2020; Astesana et al., 2021). Ratio measurements were performed and plotted on-line every 3 s. All experiments were conducted at RT (22–24°C).

### 2.12. Statistical analysis

The data were analyzed using the statistical software GraphPad Prism 9.1.2.^1^ Data obtained from flow cytometry experiments are presented as mean ± SE. Differences were considered statistically significant when *p* ≤ 0.05. The statistical tests used for each experiment are reported in figure legends. All the Ca^2+^ signaling data were obtained from at least three different batches of ClK cells. Each trace shown is the average of the Ca^2+^ tracings recorded from multiple cells displaying a similar Ca^2+^ activity within the same field of view. The peak amplitude of ATP-induced intracellular Ca^2+^ release and entry were measured by evaluating the difference between the F340/F380 ratio at the peak of the Ca^2+^ response and the mean F340/F380 ratio of 1 min baseline recording before agonist addition. Pooled data are presented as mean ± SE, and statistical significance (*p* ≤ 0.05) was evaluated by One-Way ANOVA analysis. The number of cells measured for each experimental condition is indicated in, or above, the corresponding bar histogram.

## 3. Results

### 3.1. Generation of immortalized EGCs

**Figure 1:**
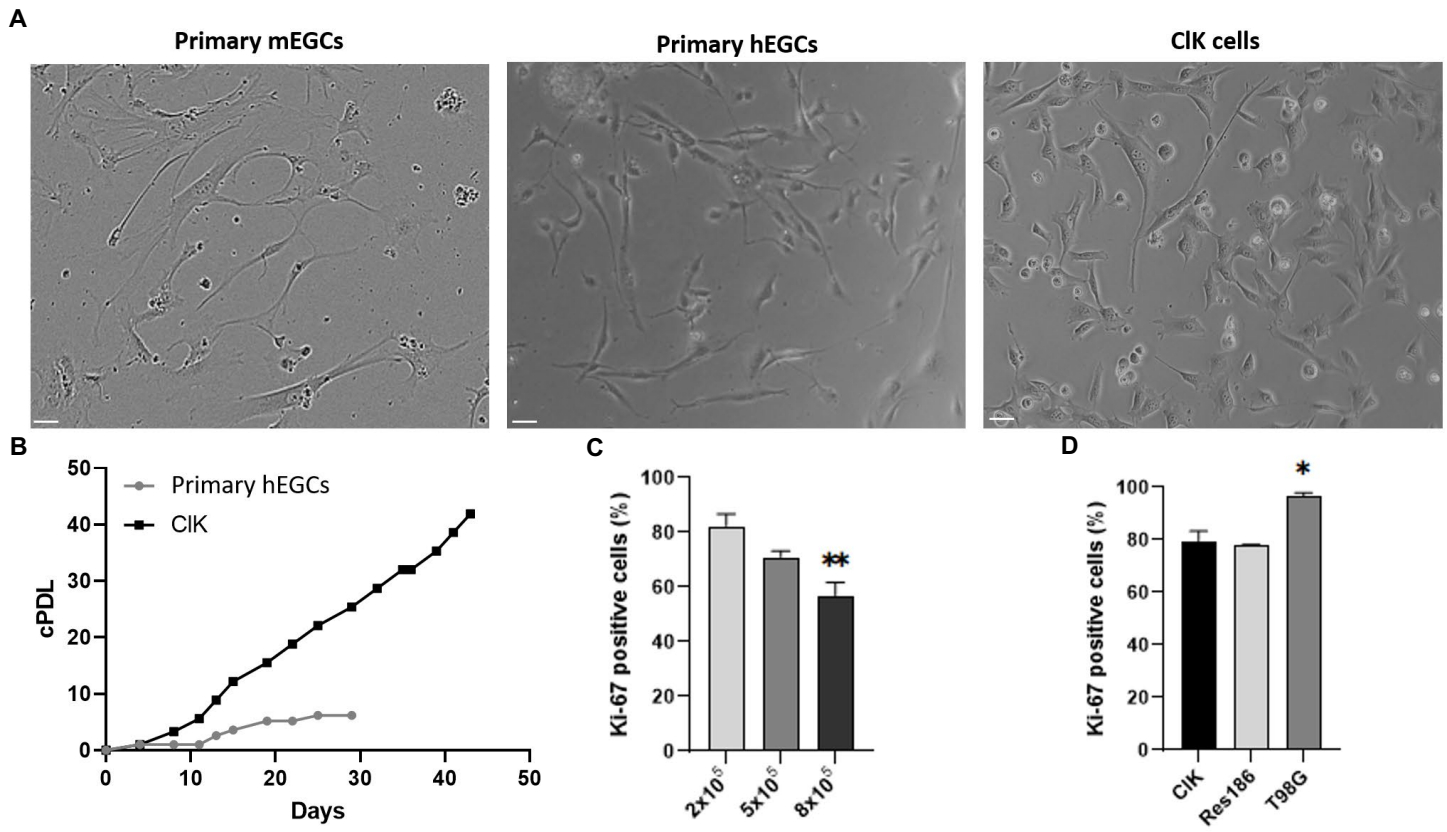
Comparative analysis of ClK morphology and proliferation. **(A)** Optical microscope analysis (brightfield) of primary hEGCs (2 days post-isolation), primary mEGCs (2 days post-isolation), and ClK cells (p21). Size bars (= 10 μm) are reported. **(B)** Cumulative population doublings (cPDL) of primary hEGCs and ClK cells following a time-kinetics. Mock-infected control cells died 10 days after infection (data not shown). cPDL values are reported in Supplementary Table S3. **(C)** Percentage of Ki-67 positive ClK cells at different cells concentrations (i.e., 2–5-8×10^5^). On X-axis is reported the number of cells whereas on Y-axis the percentage of cells positive to Ki-67. (^**^) indicates *p* < 0.01, ANOVA One-Way compared with the lower concentration. **(D)** Comparison of the percentages of Ki-67 ClK positive cells with human low- (Res186) and high-grade astrocytoma (T98G) established cell lines, all at 2×10^5^. (^*^) indicates *p* < 0.05, ANOVA One-Way, compared with ClK cells. Experiments were performed on three independent biological replicas. Corresponding cytofluorimetric plots are reported in Supplementary Figure S2.

Primary hEGCs were isolated from a human intestinal biopsy and then transduced with lentiviral vectors carrying 33 different genes (Supplementary Table S1), as previously described (Lipps et al., 2018). A total of 30 clones were selected according to their proliferative rate and expanded for two months. Among these, one relevant clone (hereafter referred to as ClK) was selected for further investigations according to its morphological characteristics and growth rate. PCR analysis on ClK cells identified 8 integrated transgenes, i.e., *Core*, *BMI1*, *E6*, *E7*, *ID1*, *MYC*, *Nanog,* and *REX* (Supplementary Figure S1). As highlighted in Figure 1A, ClK cells showed a similar morphology to primary mouse (mEGCs) and human (hEGCs) ones. Subsequently, the cumulative population doubling/days (cPDL) was calculated for ClK cells and compared with that of primary hEGCs. As shown in Figure 1B, the selected clone showed increasing cPDL compared to primary hEGCs, which reached a plateau after 19 days. Furthermore, primary hEGC growth stopped after 29 days and became senescent, thus leading to the primary cell culture death after subculturing. ClK cells have been already expanded until passage 35, and preliminary analyses confirmed they maintain their morphology as well as the expression of the glial markers GFAP and S100β. Moreover, flow cytometry assays showed that also at this passage ClK cells have low amount of intracellular ROS levels as well as no event of mitochondrial depolarization (Supplementary Figure S2).

Basing on the assayed proliferation rates and accordingly to technical recommendations (Lipps et al., 2018), ClK cells at an early culture passage (p21) were then used for subsequent morphological, cytogenetic, molecular, and physiological analyses. Proliferation levels of ClK clone was then studied through flow cytometry analysis of the Ki-67 expression. Specifically, ClK cells were seeded at different concentrations for 24 h (2–5-8 × 10^5^ in a multiwell-6) and then compared with the same concentrations of two established glioma cell lines at different malignancy grades, respectively Res186 (WHO grade I) and T98G (IV). As reported (Figure 1C), the percentage of Ki-67 positive cells significantly decreased in a concentration-dependent manner. Moreover, the amount of ClK-positive cells was comparable with those detected in Res186 cells, while they were significantly lower compared with those scored in T98G cells (Figure 1D). Furthermore, the percentage of Ki-67-positive cells in the T98G cell line did not show variations due to the cell confluence (Supplementary Figure S3).

### 3.2. Ultrastructural analysis and mitochondrial membrane potential (ΔΨm) measurement

**Figure 2:**
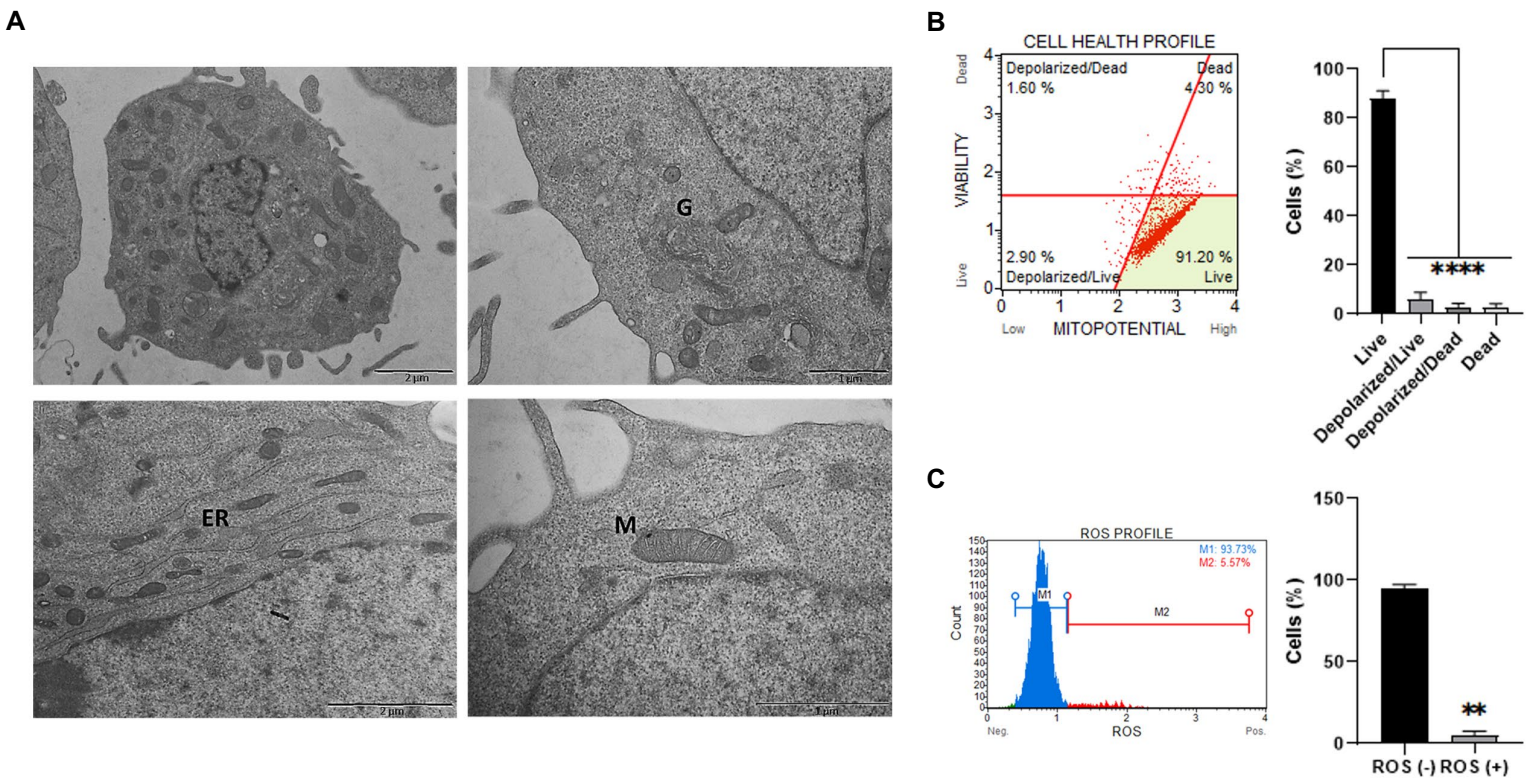
Ultrastructural and functional characterization of ClK cells. **(A)** TEM analysis of EG ClK cells (p21). M: mitochondria; G: Golgi apparatus; ER: endoplasmic reticulum. Scale bars (1–2 μm) are reported. **(B)** Cytofluorimetric plot and relative histogram reporting the mitochondrial depolarization percentages of ClK cells. Events collected: 2000 cells. Experiments were performed on three independent biological replicates. Data are reported as mean ± SE. On Y-axis is reported the number of cells expressed as percentages (%). (^****^) indicate *p* ≤ 0.0001, ANOVA One-Way. **(C)** Cytofluorimetric ROS profile plot (M1 gate: ROS-; M2 gate: ROS+) and relative histograms are reported. Events collected: 2,000 cells. Experiments were performed on three independent biological replicates. Histograms data are represented as mean ± SE. On Y-axis is reported the number of cells expressed as percentages (%). (^**^) indicates *p* ≤ 0.01, *t*-test.

Cellular and organelles morphology was investigated through ultrastructural analysis by TEM. Particularly, as reported in Figure 2A, integrity of mitochondrial shape and internal membrane crests was verified. Moreover, no significant alterations were detected in the other cellular organelles, such as Golgi apparatus and endoplasmic reticulum. Then, the mitochondrial membrane potential (ΔΨm) was studied since alterations in mitochondria functionality can affect the ΔΨm and, as a consequence, cell viability (Kroemer and Reed, 2000). Specifically, mitochondrial depolarization was assessed through flow cytometry (Figure 2B). As reported, a statistically significant low percentage of cells characterized by depolarized events were detected (i.e., depolarized live cells = 2.90%; depolarized dead cells = 1.90%) in the overall cell population. Lastly, basal intracellular reactive oxygen species (ROS) levels were investigated using flow cytometry analysis. Increased ROS levels are indeed typical of transformed or pathological cells, produced by a large variety of internal and environmental factors (Yang et al., 2018). As shown (Figure 2C), ClK cells were characterized by relatively low intracellular levels of ROS (i.e., 5.57%). Moreover, as reported in Supplementary Figure S4, ClK cells showed intracellular ROS levels similar to human primary fibroblasts, while human primary retinal endothelial cells as well as glioma cell lines (i.e., Res186 and T98G) showed higher basal ROS levels compared with ClK cells.

### 3.3. Cytogenetic analysis

**Figure 3:**
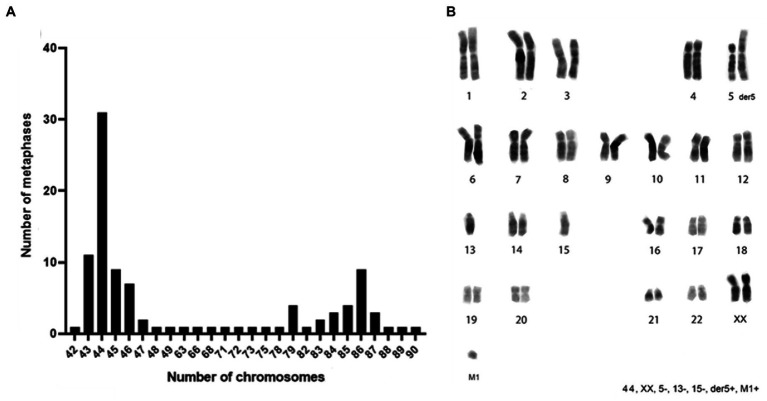
Chromosomes distribution and karyotype reconstruction of ClK clone. **(A)** Distribution of the number of chromosomes counted in 100 metaphases and **(B)** consensus karyotype of the ClK clone. The related values are reported in Supplementary Table S4.

Considering the transgenes integration events, ClK cells were selected for the cytogenetic analysis to verify the genome integrity and the presence of chromosomal rearrangements. The chromosome number was counted in a sample of 100 metaphase spreads. As reported in Figure 3A, heteroploidy was observed in the analyzed cell population. Notably, two main cell populations were observed: one near-diploid (31% of the cells, chromosome number 2*n* = 44) and one near-tetraploid (9% of the cells, chromosome number 2*n* = 86). Karyotype reconstruction was carried out in a sample of 20 metaphase spreads belonging to the most represented cell population (2*n* = 44), using reverse DAPI banding (Figure 3B). The analysis of the reconstructed karyotypes showed a monosomy of chromosomes 5, 13, and 15 as well as the presence of a big metacentric derivative chromosome comparable in size to chromosome 1. A small chromosome fragment (marker M1) was also identified in single copy in 39% of the analyzed metaphase spreads. In particular, the M1 marker was present in 19% of the cells with a total chromosome number of 44 as well as in 19% of the metaphases presenting 86 chromosomes (Supplementary Figure S5). A direct comparison between the large metacentric derivative chromosome and the long arm of chromosome 5 highlighted a striking similarity in their banding pattern. Therefore, it was first hypothesized that the rearranged chromosome originated from a translocation event involving the 5q arm and an unidentified chromosome fragment, leading to a partial monosomy of chromosome 5. The resulting large metacentric chromosome was indeed designated as der5.

**Figure 4:**
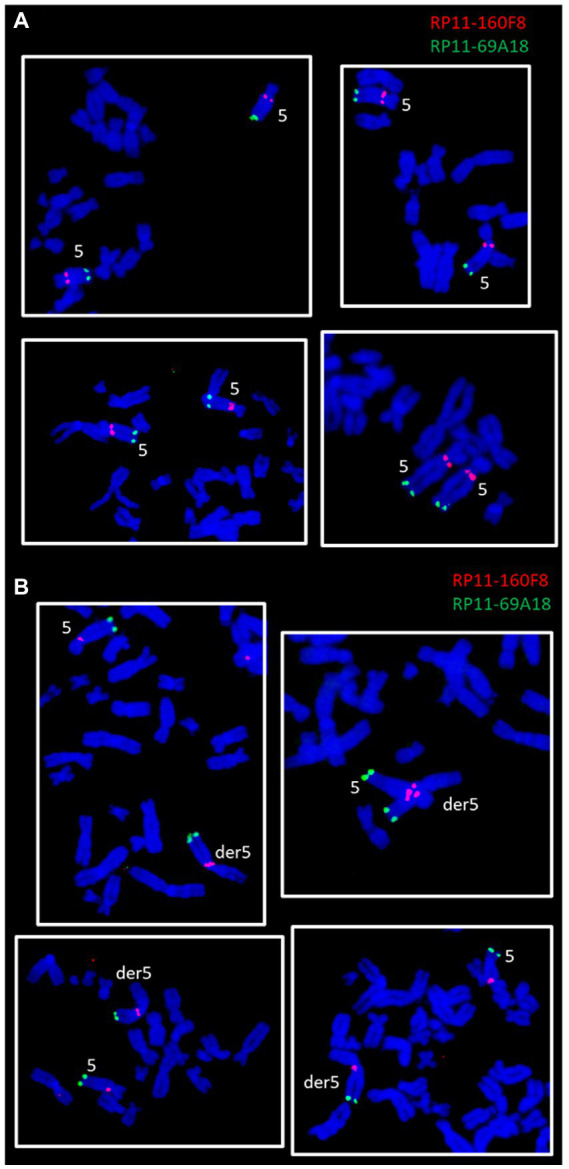
FISH experiments on ClK clone (p21) metaphases lacking **(A)** or presenting **(B)** the der5 chromosome. The panel reports two-color FISH experiments performed using a sub-centromeric (RP11-160F8, red) and a sub-telomeric (RP11-69A18, green) DNA probe specific for the long arm of chromosome 5.

The involvement of the long arm of chromosome 5 in the formation of the large derivative chromosome was verified through two-color FISH experiments using DNA probes specific for the sub-centromeric (RP11-160F8, band: q11.2, red) and sub-telomeric (RP11-69A18, band: q35.1, green) regions of chromosome 5. The chromosomal localization of the two probes was first verified by FISH on control human metaphase spreads (data not shown). Subsequently, the probes were contrastingly labelled and co-hybridized to ClK metaphases at p21. Clear fluorescence signals were detected on both normal chromosomes 5 (Figure 4A) and on the derivative marker chromosome (Figure 4B), thus confirming that the region comprised between bands 5q11.2 and 5q35.1 is involved in der5 translocation. FISH signals also provided accurate insights into the orientation of the translocated 5q fragment, which has been shown to precisely mirror the canonical arrangement reported on the original chromosome 5 (Figure 4B).

### 3.4. Assessment of the glial phenotype of ClK cells through the analysis of specific enteric glial markers

**Figure 5:**
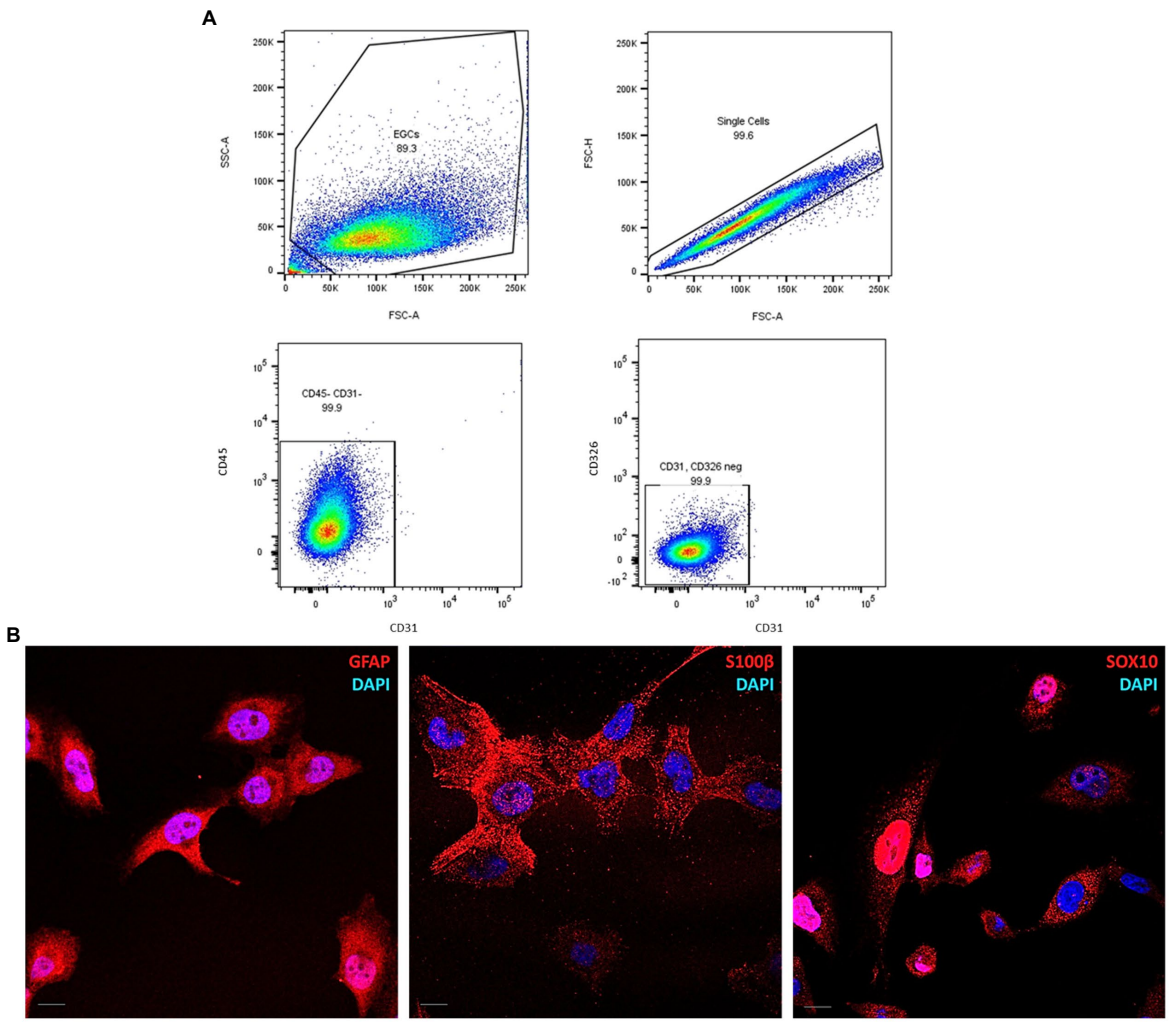
Molecular characterization of ClK cells. **(A)** Pseudocolor plots of CD31, CD45, and CD326 expression in ClK cells. Event collected: 10,000 cells. **(B)** Immunofluorescence of the enteric glial markers GFAP, SOX10, and S100β. The analysis was performed using specific anti-GFAP, anti-SOX10, and anti-S100β IgG antibodies conjugated with AlexaFluor633. Nuclei were stained with DAPI. Scale bars (=10 μm) are reported.

Flow cytometry analysis was performed to assess the homogeneity in the cell-of-origin composition of ClK clone as well as to exclude the presence of markers specific for other cell lines, such as CD31 for endothelial (Woodfin et al., 2007), CD45 for hematopoietic (Al Barashdi et al., 2021), and CD326 for epithelial cells (Keller et al., 2019). As shown in Figure 5A, no highly expressing sub-populations for the analyzed markers were observed in ClK cells. Furthermore, no positivity for CD271, a marker of mesenchymal stem cells (Álvarez-Viejo et al., 2015), was detected in the analyzed cells, since the signal was nearly overlapping the mock unstained control (Supplementary Figure S6). Finally, the expression of the enteric glial-specific makers GFAP, SOX10, and S100β (Ochoa-Cortes et al., 2016), were investigated through immunofluorescence analysis. As reported (Figure 5B), ClK cells expressed the three enteric glial markers. Furthermore, immunofluorescence analysis for the nucleoplasmatic shuttle protein SOX10 demonstrated its nuclear localization. Since EGCs express both class I and II HLA molecules on their plasma membrane (Geboes et al., 1992; da Silveira et al., 2011), basal expression of HLA molecules was studied in untreated ClK cells through immunofluorescence using a specific anti-HLA antibody. Specifically, expression levels of HLA-DQA1 were investigated in first instance due to the association with intestinal disorders (e.g., celiac disease) and drug response (Murray et al., 2007; Sazonovs et al., 2020). As reported in Supplementary Figure S7, the expression of HLA-DQA1 was confirmed by the detection of specific fluorescence signals in ClK cells although with intensity differences among cells. Specificity of all the fluorescence signals was confirmed through negative control staining. Then, HLA genotyping was performed on ClK cells. As reported in Supplementary Table S5, PCR-SSO results showed the HLA molecules expressed in the ClK clone.

**Figure 6:**
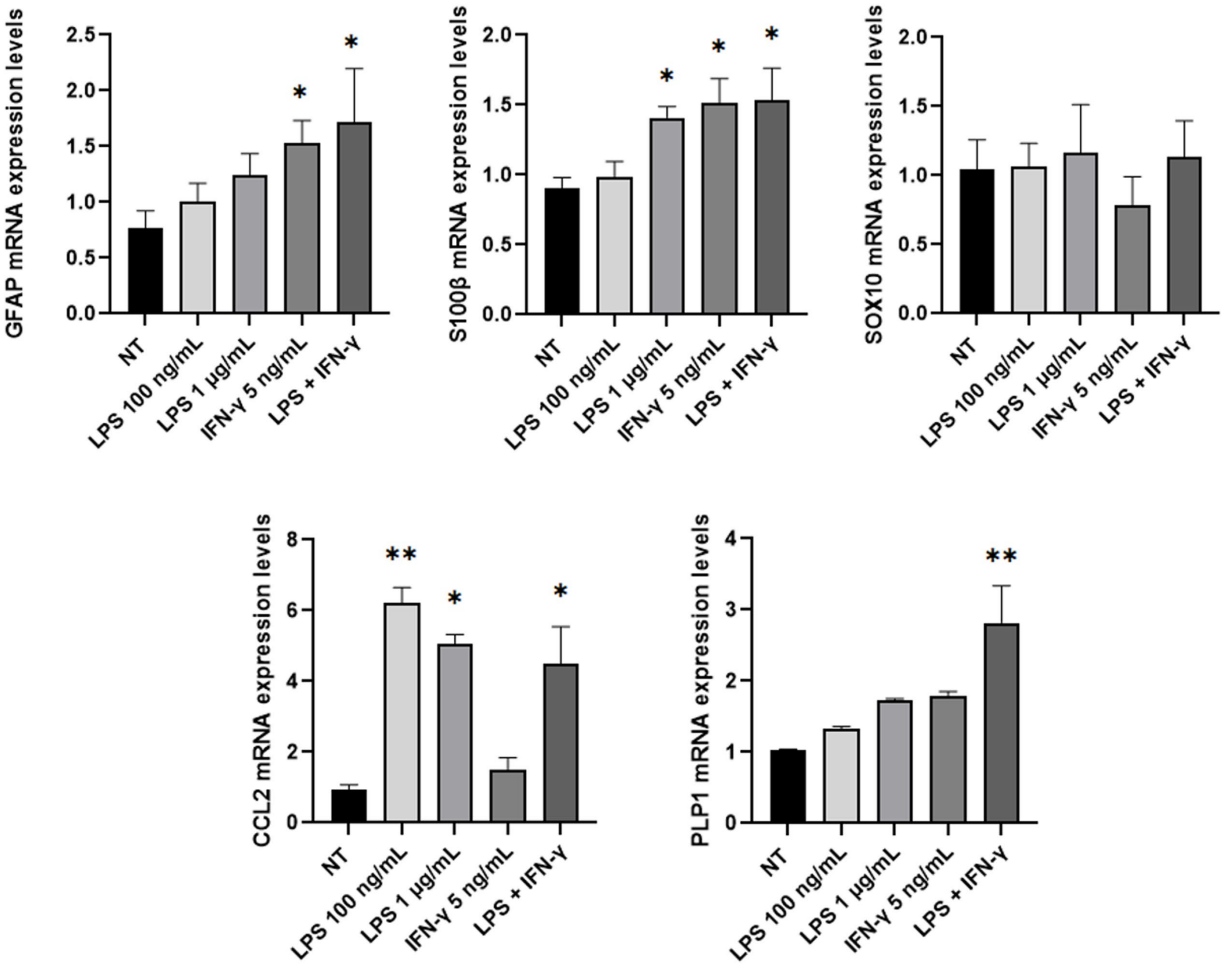
*GFAP*, *SOX10*, *S100β*, *PLP1*, and *CCL2* mRNA expression level analysis in ClK cells treated with different inflammatory stimuli for 24 h. The expression levels of the selected genes in treated cells were compared with those of non-treated (NT) cells. In the Y-axis, relative quantification values are reported. Data are reported as mean ± SE. (^*^) indicate *p* ≤ 0.05, (^**^) indicate *p* ≤ 0.01, ANOVA Kruskal-Wallis. Experiments were performed on three independent biological replicas.

Then, mRNA expression levels of *GFAP*, *SOX10*, *S100β*, *CCL2*, and *PLP1* genes were investigated through Real-Time PCR analysis after lipopolysaccharide (LPS) and/or IFN-γ treatment. Inflammatory stimuli can activate enteric glia both *in vitro* and *in vivo*, thus leading EGCs activation and consequent increase of the glial cells’ markers (von Boyen et al., 2004; Liñán-Rico et al., 2016; Rosenbaum et al., 2016; da Cunha Franceschi et al., 2017; Progatzky and Pachnis, 2022). Considering these premises, ClK cells were treated with different concentrations of LPS (i.e., 100 ng/mL and 1 μg/mL), IFN-γ (i.e., 5 ng/mL), or a combination of both (i.e., LPS 100 ng/mL + IFN-γ 5 ng/mL). As reported in Figure 6, at 24 h post-treatment *GFAP* mRNA levels showed an increasing trend in all treatments but only in presence of IFN-γ or in correspondence of LPS + IFN-γ statistically significant increases were scored compared with non-treated (NT) cells. *SOX10* did not show significant variations; conversely, a statistically significant increase in *S100β* mRNA levels was observed in the samples treated with LPS 1 μg/mL and IFN-γ. An increasing trend in *PLP1* mRNA expression was also detected in the analyzed samples, with however a statistically significant variation in correspondence of LPS + IFN-γ sample. Finally, expression levels of *CCL2* showed a statistically significant increase in all treatments except for IFN-γ.

### 3.5. ATP trigger intracellular Ca^2+^ signaling in ClK cells

**Figure 7:**
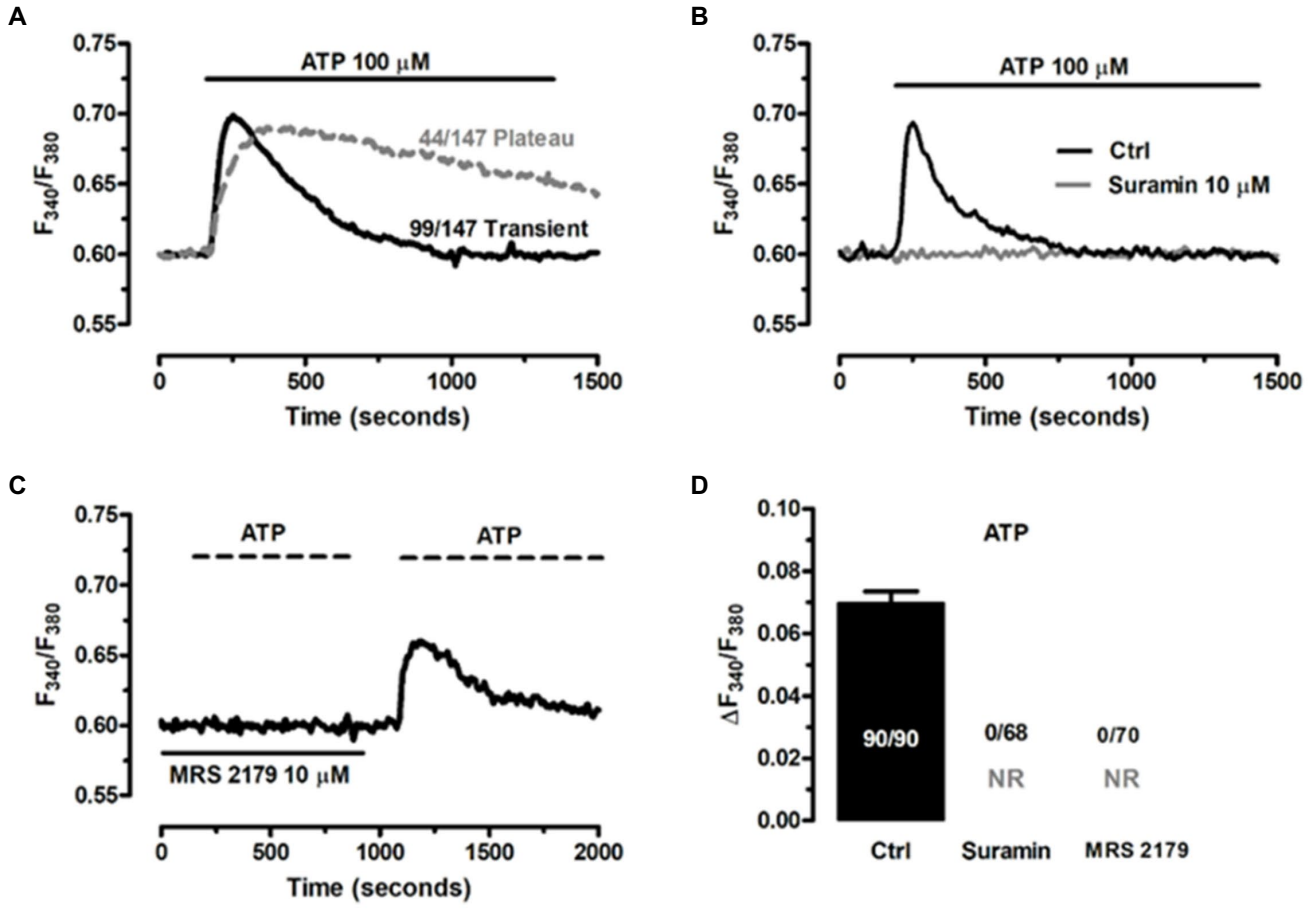
ATP induces P2Y1 receptor-mediated increase in [Ca^2+^]’ in ClK cells. **(A)** ATP (100 μM) triggered heterogeneous Ca^2+^ signals in ClK cells. **(B)** The Ca^2+^ response to ATP (100 μM) was abrogated upon preincubation with suramin (10 μM, 30 min), a non-specific P2Y receptor inhibitor. **(C)** Pretreating the ClK cells with MRS-2179 (10 μM, 30 min), a competitive antagonist of P2Y1 receptor abolished the Ca2^+^ response to ATP (100 μM), whereas the signals resumed upon washout of inhibitor. **(D)** Mean ± SE the amplitude of the Ca^2+^ response to ATP in the absence (Ctrl) and presence of P2Y receptor blockers. In panel (D); NR: no response.

An increase in intracellular Ca^2+^ concentration ([Ca^2+^]i) is the most common mechanism whereby EGCs respond to extracellular stimulation (Boesmans et al., 2013, 2019; Seguella and Gulbransen, 2021). It has long been known that neuron-to-glia communication requires neuronal release of ATP, which in turn binds to postsynaptic Gq/11 coupled P2Y1 receptors to induce an increase in [Ca^2^+]i (Gomes et al., 2009; Brown et al., 2016; Boesmans et al., 2019; Seguella and Gulbransen, 2021). Therefore, assessing ATP-induced intracellular Ca^2+^ signaling represents a widespread strategy to confirm the functionality of the ClK clone (Boesmans et al., 2013; Soret et al., 2013). According to our results, ATP (100 μM) induced intracellular Ca^2+^ signals in the majority of cells (97.3%, *n* = 147), which displayed either a transient [Ca^2+^]i spike (69.2%, *n* = 99) or a biphasic increase in [Ca^2+^]i (30.8%, *n* = 44) that comprised an initial Ca^2+^ peak followed by a prolonged plateau above the resting Ca^2+^ levels (Figure 7A). The Ca^2+^ response to ATP was abolished by suramin (Figure 7B), a non-selective P2Y receptor antagonist (Boesmans et al., 2013, 2019), and by MRS-2179 (10 μM) (Figure 7C), a selective P2Y1 receptor blocker (Boesmans et al., 2013; Mishra et al., 2016). Of note, the Ca^2+^ response to ATP resumed upon washout of MRS-2179 (Figure 7C), but not suramin (not shown). Statistical analysis of these data is reported in Figure 7D. These results showed that ATP was able to reliably evoke intracellular Ca^2+^ signals in ClK cells.

**Figure 8:**
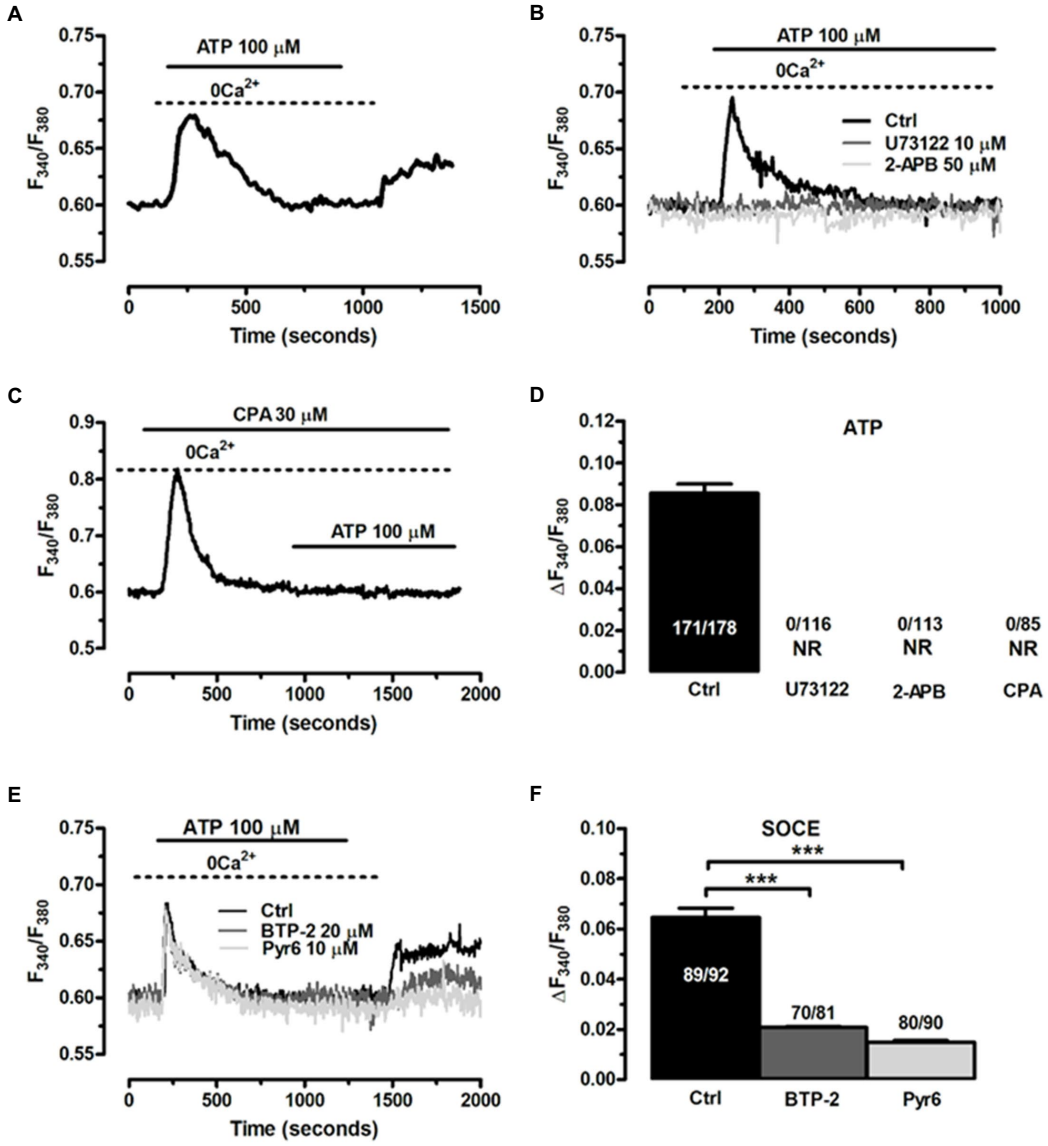
The Ca^2+^ response to ATP requires InsP_3_-induced ER Ca^2+^ release and SOCE activation in CIK cells. **(A)** Cells perfused with ATP (100 μM) in the absence of extracellular Ca^2+^ responded with a transient increase in [Ca^2+^]i, whereas restoration of extracellular Ca^2+^ caused a second elevation in [Ca^2+^]i. ATP was removed 100 s before re-addition of extracellular Ca^2+^. **(B)** ATP-induced transient Ca^2+^ release was blocked upon preincubation of the ClK cells with U73122 (10 μM, 30 min), a selective PLC inhibitor, and 2-APB (50 μM, 30 min), which blocks InsP_3_Rs under 0 Ca^2+^ conditions. ATP was administered at 100 μM. **(C)** Pharmacological depletion of the ER Ca^2+^ pool with CPA (30 μM) in the absence of extracellular Ca^2+^ (0 Ca^2+^) inhibits the Ca^2+^ release evoked by ATP (100 μM). Note the intracellular Ca^2+^ transient evoked by CPA due to the passive leakage of ER Ca^2+^ in the cytosol, as more widely described in the text. **(D)** Mean ± SE of the amplitude of ATP-induced intracellular Ca^2+^ release under the designated treatments. **(E)** ATP-induced extracellular Ca^2+^ entry was dramatically reduced by selectively blocking SOCE with BTP-2 (20 μM, 20 min) or Pyr6 (10 μM, 10 min). **(F)** mean ± SE of the amplitude of the peak Ca^2+^ entry by ATP under the designated treatments. In panel d; NR: no response. One-way ANOVA analysis was used for Statistical comparison. In Panel F: (^***^) *p* ≤ 0.005.

Based upon the evidence obtained from EGCs isolated from different animal species, the Ca^2+^ response to ATP in ClK cells was likely to be triggered by inositol-1,4,5-trisphosphate (InsP3)-dependent Ca^2+^ release from the endoplasmic reticulum (ER) (Verkhratsky and Parpura, 2014). Accordingly, in the absence of extracellular Ca^2+^ (0 Ca^2+^), ATP (100 μM) evoked a transient increase in [Ca^2+^]i, which was consistent with the depletion of endogenous Ca^2+^ stores (Figure 8A). The magnitude of the increase in [Ca^2+^]i peak was significantly lower (*p* < 0.001) compared to the Ca^2+^ response induced by ATP in the presence of extracellular Ca^2+^ (0.078 ± 0.004, *n* = 220, vs. 0.093 ± 0.004, *n* = 179). Interestingly, the subsequent re-addition of extracellular Ca^2+^ to the perfusate induced a second, discernible peak in [Ca^2+^]i that was due to extracellular Ca^2+^ entry in 167 out of 227 cells (Figure 8A). The agonist, i.e., ATP, was removed 100 s before restitution of external Ca^2+^ (Figure 8A). Therefore, ATP-induced extracellular Ca^2+^ entry did not occur either through ionotropic P2X receptors or second messengers operated channels (SMOCs) and was likely to be mediated by store-operated Ca^2+^ entry (SOCE), a ubiquitous Ca^2+^ entry route that only requires the previous depletion of the ER Ca^2+^ pool (Bird et al., 2008; Negri et al., 2020) and sustains the Ca^2+^ response to ATP in enteric glia (Sarosi et al., 1998). To further support this evidence, we adopted an established pharmacological approach to inhibit InsP3-induced ER Ca^2+^ release and SOCE. Blocking phospholipase Cβ (PLCβ) with U73122 (10 μM, 20 min) (Figures 8B,D; Zhang et al., 2003), and inhibiting InsP3 receptors (InsP3Rs) with 2-Aminoethyl diphenylborinate (2-APB; 50 μM, 20 min) (Figures 8B,D) suppressed ATP-induced ER Ca^2+^ mobilization (Muller and Taylor, 2017; Astesana et al., 2021). In addition, the intracellular Ca^2+^ response to ATP was abolished by interfering with the activity of Sarco-Endoplasmic Ca^2+^-ATPase activity (SERCA) with cyclopiazonic acid (CPA) (Dragoni et al., 2014; Figures 8C,D). SERCA represents the Ca^2+^ pump that sequesters cytosolic Ca^2+^ into ER lumen and is, therefore, responsible for maintaining ER Ca^2+^ concentration ([Ca^2+^]ER) (Verkhratsky and Parpura, 2014). CPA blocks SERCA activity, thereby causing a transient increase in [Ca^2+^]i that results from passive leakage of Ca^2+^ from the followed by the recovery of to the baseline due to the concerted activity of Na^+^/Ca^2+^ exchanger, plasma membrane Ca^2+^-ATPase and mitochondria (Bird et al., 2008; Negri et al., 2020). As shown in Figure 8C, CPA (30 μM) caused a transient rise in [Ca^2+^]i under 0 Ca^2+^ conditions, which reflected ER Ca^2+^ depletion. Indeed, after 20 min in the presence of CPA, ATP failed to elevate [Ca^2+^]i, thereby confirming that InsP3-induced ER Ca^2+^ release was the mechanism responsible for the initial Ca^2+^ peak. SOCE is the Ca^2+^ entry pathway activated upon InsP3-induced reduction in [Ca^2+^]ER to refill the endogenous Ca^2+^ reservoir and prolong the Ca^2+^ response to extracellular stimuli in non-excitable cells (Prakriya and Lewis, 2015), including glial cells (Verkhratsky and Parpura, 2014). The role of SOCE in ATP-induced extracellular Ca^2+^ entry in ClK cells was examined by exploiting the “Ca^2+^ add-back” protocol described in Figure 8A in the absence and in the presence of two established blockers of Orai1 (Zhang et al., 2003), which provides the pore-forming subunit of store-operated Ca^2+^ channels (SOCs) in glial cells (Toth et al., 2019). ATP-induced extracellular Ca^2+^ entry in ClK cells was significantly reduced (*p* < 0.005) by pretreating the cells with BTP-2 (20 μM, 20 min) or Pyr6 (10 μM, 20 min) (Figures 8E,F), while ER Ca^2+^ release was not impaired (Figures 8E,F), thereby confirming the selectivity of each drug towards SOCE. These findings, therefore, confirm that ATP, the main mediator of the neuron-to-glia communication in the ENS, is able to activate the ClK clone through an increase in [Ca^2+^]i, which arises downstream of P2Y1 receptors and requires InsP3-dependent ER Ca^2+^ release and SOCE.

Finally, we assess whether other neurotransmitters were able to activate ClK cells by increasing [Ca^2+^]i. Supplementary Figure S8 shows that acetylcholine (Ach, 10 μM) (**Panel A**), serotonin (5-hydroxytryptamine or 5-HT, 10 μM) (**Panel B**), and glutamate (100 μM) (**Panel C**) elicited intracellular Ca^2+^ signals that could adopt either a transient or an oscillator pattern. Therefore, as commonly reported for EGCs (Boesmans et al., 2013, 2019; Seguella and Gulbransen, 2021), intracellular Ca^2+^ signalling is also crucial to the activation of ClK cells by enteric neurotransmitters.

## 4. Discussion

Interest in EGCs biology has increased in recent years due to the discovery of their key roles in intestinal homeostasis. However, the lack of proper methods to isolate and manipulate these cells has hampered their characterization from both phenotypical and functional points of view (Wang et al., 2018). Biological studies of EGCs are also challenging since these cells *in vivo* constitute a heterogeneous population according to their intestinal topology. Furthermore, the lack of a suitable human model still represents an important limitation in functional studies of EGCs. Nowadays, EGCs can be isolated from different organisms (e.g., guinea pig, mouse, rat and human) although with several technical difficulties (Soret et al., 2013). Another problem is represented by the tendency of these cells to de-differentiate *in vitro* in both neurons and non-myelinating Schwann cells (Jessen and Mirsky, 1983). De-differentiation of EGCs is also enhanced by the time-requiring steps necessary for the purification and establishment of these cells from the intestine (Soret et al., 2013).

In this work, a validated transduction approach was used to generate immortalized human EGC clones. This method generates immortalized cell lines that closely resemble their original precursors in a rapid and reliable way (Lipps et al., 2018). Compared to other currently available techniques (e.g., iPS differentiation or trans-differentiation), this protocol avoids the risk of obtaining heterogeneous cell lines or cells with low proliferation rates. Moreover, contrary to other techniques such as the reactivation of human telomerase reverse transcriptase (hTert), this transduction method can be used virtually with every cell type (Lipps et al., 2018).

As reported in this contribution, an immortalized EGC line (referred to as ClK clone) was obtained starting from a human surgery of the myenteric plexus of a histologically normal intestinal tissue with no aberrations. Compared to primary cells that reached growth plateau and arrest (respectively, 19- and 29-days post-isolation), increasing cPDL revealed that ClK cells acquired an actively proliferating phenotype. Notably, flow cytometry analysis of Ki-67, a well-known marker of cell proliferation (Sun and Kaufman, 2018), showed a significant reduction in the Ki-67 positive sub-population in ClK cells compared with the high-grade astrocytoma T98G cell line as well as a decreasing trend according to cell confluence. These data suggested a possible contact inhibition of proliferation for ECGs, a characteristic typical of noncancerous cells that is generally lost in transformed cells (Pavel et al., 2018). This hypothesis was also supported by the evidence collected on other developed immortalized cell lines derived with the same transduction protocol that did not show the ability to develop tumors *in vivo* (Lipps et al., 2018). Furthermore, TEM and flow cytometry analyses showed no evident structural/morphological variations both in the topology and in the intracellular compartments of ClK cells, as well as perturbations of mitochondrial membrane potential. Moreover, ROS intracellular levels were detected in less than 6% of the analyzed cells. As demonstrated by subsequent flow cytometry and immunofluorescence analyses, ClK cells express the EGCs-specific markers (i.e., GFAP, SOX10, and S100β) (Hoff et al., 2008; De Giorgio et al., 2012), that are associated with mature EGCs and are implicated in several biological pathways (Bush et al., 1998; Rühl et al., 2001b; von Boyen et al., 2004; Cirillo et al., 2011b; da Cunha Franceschi et al., 2017), but no expression was observed for endothelial, hematopoietic, and epithelial specific markers (Woodfin et al., 2007; Álvarez-Viejo et al., 2015; Keller et al., 2019; Al Barashdi et al., 2021), underlining the lack of contamination by other cell types in the isolated EGCs. Notably, the analysis of the mRNA expression levels of *GFAP*, *SOX10*, *S100β*, *PLP1*, and *CCL2* EGC markers (von Boyen et al., 2004; Liñán-Rico et al., 2016; Rosenbaum et al., 2016; da Cunha Franceschi et al., 2017; Progatzky and Pachnis, 2022) demonstrated the capability of ClK cells to react against inflammatory stimuli, as expected for EGCs. Considering emerging evidence regarding HLA expression by EGCs (Koretz et al., 1987; Geboes et al., 1992; Denzer et al., 2000; da Silveira et al., 2011) immunofluorescence as well as HLA genotyping through reverse PCR-SSO were performed. The analyzes revealed that ClK cells expressed HLA-DQA1 molecule and identified a narcolepsy-cataplexy associated allele (namely, HLA-DQB1*0602) (Lin et al., 2001), whereas no alleles associated with intestinal diseases (e.g., celiac disease, inflammatory bowel diseases) or co-morbidities were found (e.g., diabetes).

An accurate cytogenetic analysis of ClK clone was carried out to investigate the presence of structural and numerical chromosome changes, which have been shown to frequently occur *in vitro* upon immortalization processes (Goymer, 2008). Firstly, the distribution of the chromosome number per metaphase was assessed. As reported, ClK cells showed a modal karyotype of 44 chromosomes (2*n* = 44) together with one recurrent chromosomal rearrangement, as already described for other cell lines obtained with this technology (Lipps et al., 2018). Notably, the distribution of the chromosome number showed a tendency to acquire a near-tetraploid arrangement (4n), as suggested by the second modal value detected (2*n* = 86). In the majority of the analyzed metaphases, a big metacentric derivative chromosome (der5), as well as a small chromosome fragment (M1), were also detected. As M1 occurs in a single copy in a considerable number of metaphases (38%), it may be hypothesized that it contains a functional centromere. Nevertheless, immunofluorescence experiments targeting the centromere-specific histone variant CENP-A are further required to validate this assumption (Quénet and Dalal, 2012). The origin of the large metacentric rearranged chromosome has been also objecting of deepen the analysis. According to the preliminary results obtained from the reverse DAPI banding, it was first hypothesized that this chromosome contained the entire 5q arm. This assumption was later confirmed by two-color FISH experiments using DNA BAC probes specific for the sub-centromeric and sub-telomeric regions of the long arm of chromosome 5. The results obtained clearly confirmed the involvement of the 5q arm in the formation of the der5 chromosome. In light of these data, the consensus karyotype of the ClK cell line at p21 was designated as 44, XX, 5-, 13-, 15-, der5+, M1+/−. At present, the origin of the other translocated arm remains unidentified. Additional FISH experiments might be required to fully dissect der5 composition. Given the complete monosomy of chromosomes 13 and 15 detected in the modal karyotype, a likely hypothesis is that the long arm of their missing homolog may be involved in this rearrangement. Future perspectives might include the analysis of ClK karyotype at higher culture passages to provide clear hints into its evolution over time. However, as already reported, cell lines obtained with this transduction approach showed few ploidy changes after extended cultivation, suggesting a relative stability of the karyotype (Lipps et al., 2018).

ATP represents one of the most ubiquitous mediators of intercellular communication between enteric neurons and EGCs (Seguella and Gulbransen, 2021). Accordingly, ATP can be released either by direct stimulation of intrinsic nerves (Gulbransen and Sharkey, 2009; Gulbransen and Sharkey, 2012) or extrinsic cholinergic nerve fibers, which may co-release acetylcholine and ATP (Gulbransen et al., 2010; Gulbransen and Sharkey, 2012). ATP-evoked intracellular Ca^2+^ signals in EGCs integrate neuronal activity in the myenteric plexus and are instrumental to coordinate patterns of contractive activity in the GI (Gulbransen and Sharkey, 2009; Seguella and Gulbransen, 2021). The Ca^2+^ response to ATP in cultured EGCs from multiple sources (e.g., human, mouse, rat, guinea pig) is operated by the Gq/11 coupled P2Y1 receptors (Gomes et al., 2009; Brown et al., 2016; Boesmans et al., 2019; Seguella and Gulbransen, 2021), triggered by InsP3-induced ER Ca^2+^ mobilization (Grubišić and Gulbransen, 2017) and prolonged by SOCE (Sarosi et al., 1998). We found that ATP could evoke two different patterns of intracellular Ca^2+^ signals in ClK cells: a transient increase in [Ca^2+^]i that returned to the baseline despite the continuous exposure to the agonist, and a biphasic Ca^2+^ signal that persisted as long as ATP was present in the perfusate. Prolonged stimulation with ATP has previously been shown to induce a biphasic increase in [Ca^2+^]i in guinea pig EGCs (Kimball and Mulholland, 1996; Gulbransen and Sharkey, 2009), whereas variable waveforms (transient vs. sustained) were evoked by ATP in mouse (Boesmans et al., 2019) and human (Boesmans et al., 2013) EGCs. For instance, besides inducing a transient Ca^2+^ spike, ATP could evoke a biphasic increase in mouse EGCs (Boesmans et al., 2019) and multiple Ca^2+^ spikes in human ones (Brown et al., 2016). Likewise, ATP evokes intracellular Ca^2+^ signals of different kinetics also in mouse brain astrocytes (James et al., 2011; Tang et al., 2015; Taheri et al., 2017) and this heterogeneity in ATP-induced Ca^2+^ waveforms may be due to the different extent of either ATP degradation by ectonucleotidases (Berra-Romani et al., 2004) or SOCE activation (Taheri et al., 2017). Thus, the diversity of ATP-induced intracellular Ca^2+^ signals in ClK cells reflected the variability previously reported in both enteric and brain glial cells. The following evidence indicated that the Ca^2+^ response to ATP in the ClK clone is operated by P2Y1 receptors, triggered by InsP3-induced ER Ca^2+^ release, and sustained by SOCE. Firstly, the onset of the Ca^2+^ signal was abrogated by blocking P2Y1 receptors with either suramin or MRS-2179 (Boesmans et al., 2013, 2019; Mishra et al., 2016). Secondly, the Ca^2+^ response to ATP arose in the absence of extracellular Ca^2+^, although the plateau phase was abolished and the duration of the Ca^2+^ signal was significantly shorter as compared to ClK cells displaying a transient [Ca^2+^]i rise also in PSS. Thus, mobilization of the endogenous Ca^2+^ store is required to trigger ATP-induced intracellular Ca^2+^ signals in these cells. Thirdly, ATP-induced intracellular Ca^2+^ release was abrogated by preventing InsP3 production with U73122 and by directly blocking InsP3Rs with 2-APB (Zhang et al., 2003; Astesana et al., 2021). Moreover, ATP failed to induce a detectable increase in [Ca^2+^]i upon depletion of the ER Ca^2+^ store with the SERCA inhibitor, CPA, which provides a common pharmacological tool to cause a drop in [Ca^2+^]_ER_ in EGCs (Kimball and Mulholland, 1996; Zhang et al., 2003). Fourthly, extracellular Ca^2+^ entry was required to achieve the full Ca^2+^ peak and to sustain the plateau in ClK cells displaying a long-lasting Ca^2+^ response. The “Ca^2+^ add-back” protocol revealed that, after the initial depletion of the InsP3-sensitive ER Ca^2+^ pool, ATP induced the influx of extracellular Ca^2+^. As widely discussed elsewhere (Bird et al., 2008; Negri et al., 2020), Ca^2+^ entry did not require the presence of the agonist in the perfusate, which is the hallmark of SOCE activation. In agreement with this hypothesis, ATP-evoked extracellular Ca^2+^ was strongly reduced by BTP-2 and Pyr6, two pyrazole-derivatives that selectively block Orai1, the pore-forming subunits of SOCs in glial cells (Toth et al., 2019). Previous contributions have shown that SOCE prolonged the Ca^2+^ response evoked in guinea pig EGCs by ATP (Sarosi et al., 1998) and endothelin-1 (Zhang et al., 1997), whereas this is the first time that SOCE activation is reported in a human-derived EGCs. Whereas the full characterization of the Ca^2+^ handling machinery awaits future investigation, this preliminary evidence demonstrates that the ClK cells are able to generate a functional Ca^2+^ signal in response to ATP, one of the main mediators of neuron-to-glia communication in the ENS. Furthermore, ClK cells displays intracellular [Ca^2+^] signals also in response to other enteric neurotransmitters, such as Ach, 5-HT, and glutamate, which are known to activate EGCs through an increase in [Ca^2+^]i (Boesmans et al., 2013, 2019; Seguella and Gulbransen, 2021).

In conclusion, the isolation and characterization of the described human immortalized EGCs might represent a potential *in vitro* valuable tool for many applications including drug discovery as well as for disease understanding and personalized medicine approaches.

## Data availability statement

The raw data supporting the conclusions of this article will be made available by the authors, without undue reservation.

## Ethics statement

The studies involving human participants were reviewed and approved by Tissue Solutions (Glasgow, Scotland, United Kingdom). The patients/participants provided their written informed consent to participate in this study.

## Author contributions

GB, FrM, FeM, ER, TM, and SC: conceptualization. LZ, AV, KN, PF, CC, RC, IS, FC, DA, and FeM: investigation. FeM, PF, and KN: data analysis. FeM: writing-original draft preparation. RC, VDS, LvB, GB, FrM, ER, PF, and SC: writing-review and editing. FrM, FeM, ER, TM, GM, and SC: supervision. FeM and SC: project administration. MB, FeM, and SC: funding acquisition. All authors contributed to the article and approved the submitted version.

## Conflict of interest

TM holds a patent for the immortalization technology described in this manuscript and is a shareholder of InSCREENeX GmbH, which commercializes cell lines immortalized by the described technology. TM and KN are employees of InSCREENeX GmbH.

The remaining authors declare that the research was conducted in the absence of any commercial or financial relationships that could be construed as a potential conflict of interest.

## Publisher’s note

All claims expressed in this article are solely those of the authors and do not necessarily represent those of their affiliated organizations, or those of the publisher, the editors and the reviewers. Any product that may be evaluated in this article, or claim that may be made by its manufacturer, is not guaranteed or endorsed by the publisher.

## Footnotes

^1^ https://www.graphpad.com/scientific-software/prism/
