# Supplementary material for: Cytological, molecular, cytogenetic, and physiological characterization of a novel immortalized human enteric glial cell line
Source: Front Cell Neurosci. 2023 Apr 20;17:1170309. doi: 10.3389/fncel.2023.1170309 (PMC10158601; doi:10.3389/fncel.2023.1170309)
Supplement: Supplementary file 1 [file Data_Sheet_1.docx]

Supplementary Material

# Supplementary Table 1

| **3‘ Reverse primers** | | |
| --- | --- | --- |
| **Gene** | **Sequence** | **Expected band size (bp)** |
| *ID2* | GCAGGCTGACAATAGTGGGA | 462 |
| *Fos* | GGATGATGCTGGGAACAGGA | 1054 |
| *NS1* | ATGTCCTGGAAGAGAAGGCA | 678 |
| *JUN* | TTCCTCATGCGCTTCCTCTC | 912 |
| *E2F1* | CAGGGTCTGCAATGCTACGA | 944 |
| *ßCAT* | TTATGCAAGGTCCCAGCGGT | 806 |
| *TAg* | CACCTGGCAAACTTTCCTCA | 1214 |
| *MYB* | CTTCTGGAAGCTTGTGGCCA | 780 |
| *ID3* | ATGACAAGTTCCGGAGCGAG | 453 |
| *E7* | GCCCATTAACAGGTCTTCCA | 404 |
| *E6* | ATTCGCCCTTTTACAGCTGG | 636 |
| *BCL2* | TCTGCGAAGTCACGACGGTA | 440 |
| *HOXA9* | GTTTAATGCCATAAGGCCGG | 515 |
| *BMI1* | GGGCCATTTCTTCTCCAGGT | 782 |
| *PymT* | CATCTCGGGTTGGTGTTCCA | 606 |
| *Core* | ACTTTACCCACGTTGCGCGA | 487 |
| *OCT3* | GCAAAGCAGAAACCCTCGTG | 846 |
| *KLF4* | AAGATCAAGCAGGAGGCGGT | 1084 |
| *ID1* | AGAAGCACCAAACGTGACCA | 980 |
| *MYC* | AGTGGGCTGTGAGGAGGTTT | 1001 |
| *Lmo2* | TTTCCGTCCCAGCTTGTAGT | 822 |
| *NFE2L2* | GCTGCTGAAGGAATCCTCAA | 1008 |
| *YAP1* | GCCAGGATGTGGTCTTGTTC | 950 |
| *Nanog* | TATGGAGCGGAGCAGCATTC | 935 |
| *SOX2* | CTCGCAGACCTACATGAACG | 846 |
| *RHOA* | AAGCATTTCTGTCCCAACGT | 562 |
| *EZH2* | ACTTCGAGCTCCTCTGAAGC | 1481 |
| *GLI1* | CACCACATCAACAGCGAGCA | 1144 |
| *v-MYC* | GACACCCTGAGCGATTCAGA | 1052 |
| *SUZ12* | TACCCTGGAAGTCCTGCTTG | 769 |
| *ZFP217* | CAAGAAGGGAGCACCGACAA | 1188 |
| *ID4* | CAGCAAAGTGGAGATCCTGC | 652 |
| *REX* | GCGAGCTCATTACTTGCAGG | 920 |

**Table S1.** List of the 3’ reverse primers used to identify the integrated genes. The primers sequence is reported together with the expected band size as base pairs (bp) (Lipps et al., 2018).

# Supplementary Table 2

| *GFAP* | Forward primer: 5’-CTGCTCAATGTCAAGCTG |
| --- | --- |
|  | Reverse primer: 3’-GCTGGTTTCTCGAATCTG |
| *SOX10* | Forward primer: 5’-AAGACACTAGAATCCTGACC |
|  | Reverse primer: 3’-CTGCAGAACAGGAAAATAGG |
| *S100β* | Forward primer: 5’-ACCAATATTCTGGAAGGGAG |
|  | Reverse primer: 3’-CCTCTAAGAAATGGGAAAGC |
| *PLP1* | Forward primer: 5’-AGCTGAGTTCCAAATGACCT |
|  | Reverse primer: 3’-AACGACGGTGAATGTTGAAAC |
| *CCL2* | Forward primer: 5’-CAGCCAGATGCAATCAATGCC |
|  | Reverse primer: 3’-TGGAATCCTGAACCCACTTCT |
| *GAPDH* | Forward primer: 5’-TCGGAGTCAACGGATTTG |
|  | Reverse primer: 3’-CAACAATATCCACTTTACCAGAG |

**Table S2.** List of the 5’ forward and 3’ reverse primers used to amplify the enteric glial marker genes.

# Supplementary Figure 1

| 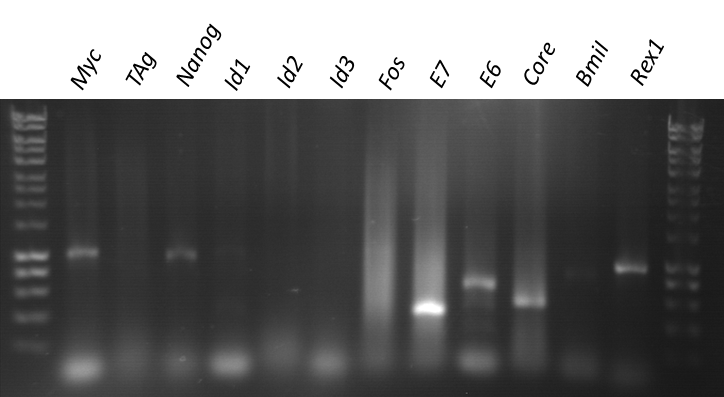 |
| --- |

**Figure S1.** Electrophoresis analysis of the integrated genes. The ladder (1 kb) is reported on both sides.

# Supplementary Table 3

| Days | *wt* | ClK |
| --- | --- | --- |
| 0 | 0 | 0 |
| 4 | 1 | 1 |
| 8 | 1 | 3.3 |
| 11 | 1 | 5.6 |
| 13 | 2.6 | 8.9 |
| 15 | 3.6 | 12.2 |
| 19 | 5.2 | 15.5 |
| 22 | 5.2 | 18.8 |
| 25 | 6.2 | 22.1 |
| 29 | 6.2 | 25.4 |
| 32 |  | 28.7 |
| 35 |  | 32 |
| 36 |  | 32 |
| 39 |  | 35.3 |
| 41 |  | 38.6 |
| 43 |  | 41.9 |

**Table S3.** Cumulative population doublings (expressed in days) of primary hEGCs and ClK cells. The clone was cultivated for more than 40 days. Primary cells stopped their growth after 29 days. Mock infected control cells died 10 days after infection (data not shown).

# Supplementary Figure 2

| 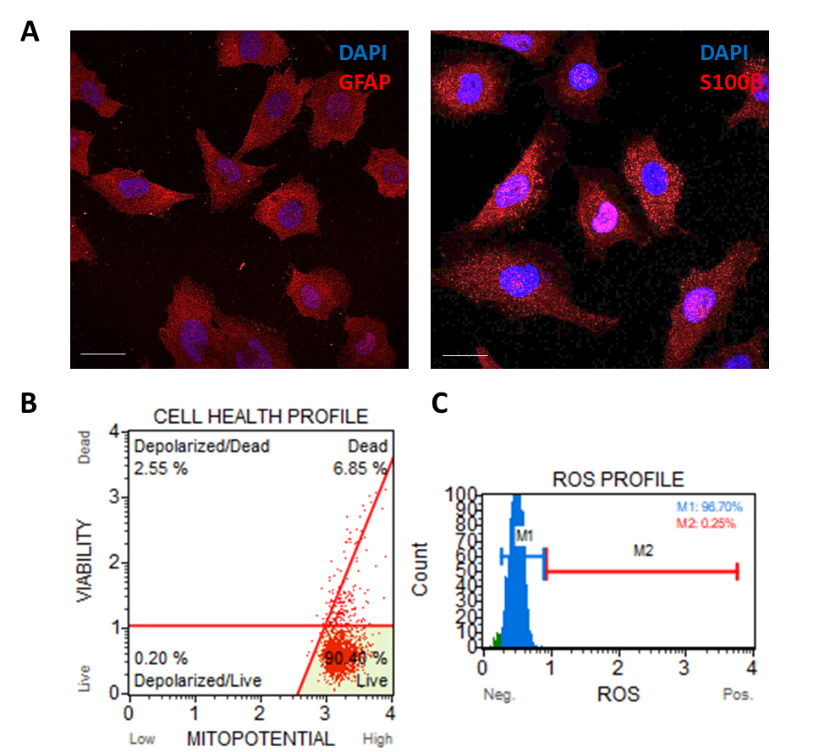 |
| --- |

**Figure S2.** Preliminary characterization of ClK cells at passage p35 through (**A**) immunofluorescence assay for the glial markers GFAP and S100β, (**B**) flow cytometry analysis of mitochondrial depolarization (events collected: 2000), and (**C**) intracellular ROS levels analysis (M1 gate: ROS-; M2 gate: ROS+).

# Supplementary Figure 3

| 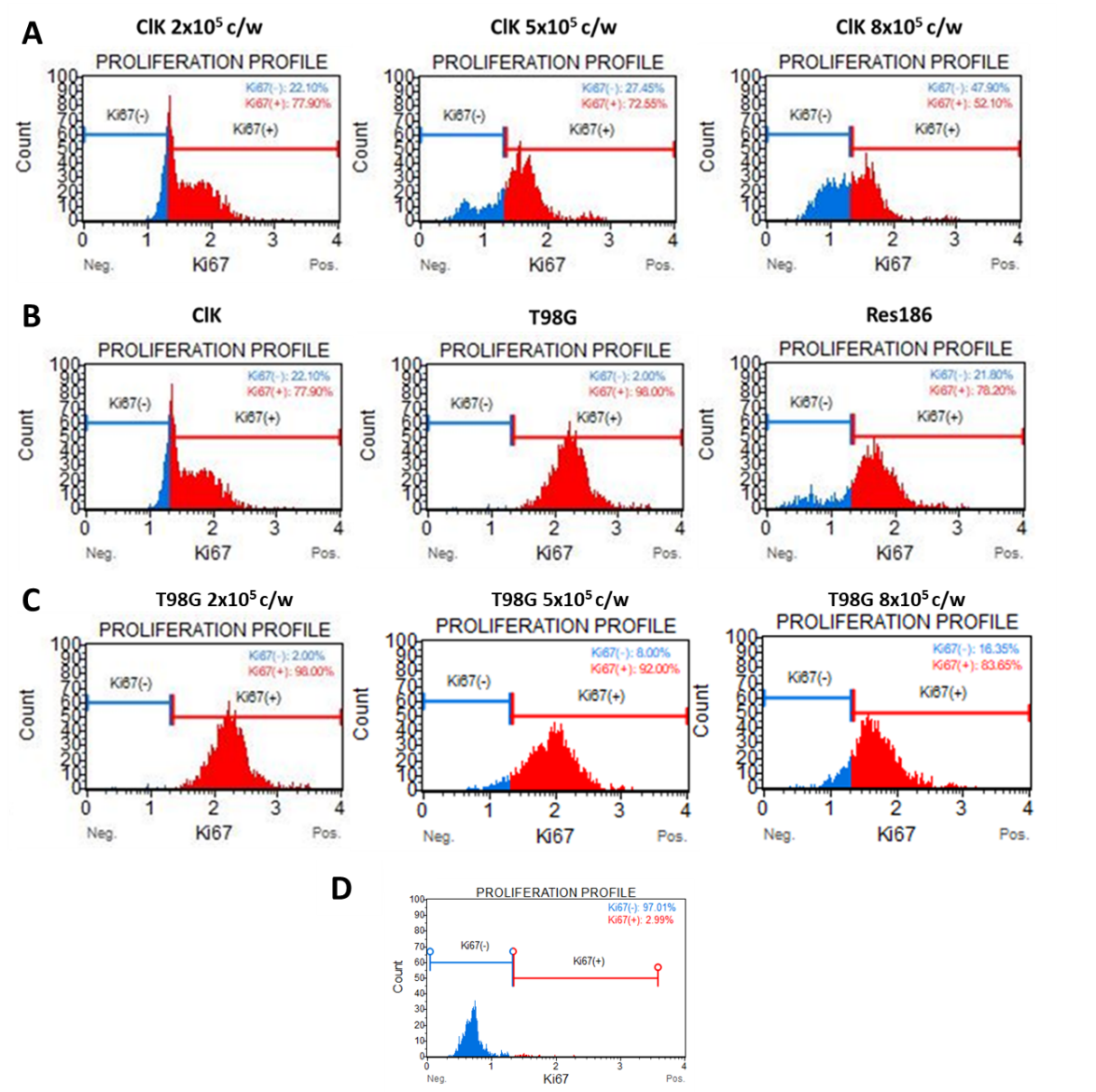 |
| --- |

**Figure S3.** Cytofluorimetric analysis of Ki-67 levels in ClK cells. (**A**) Analysis of Ki-67 expression levels in ClK at increasing confluences. Ki-67 positive cells are reported in red whereas Ki-67 negative cells in blue. Events collected: 2000. Experiments were performed in triplicate. (**B**) Analysis of Ki-67 expression levels in ClK, T98G and Res186 cells. Ki-67 positive cells are reported in red whereas Ki-67 negative cells in blue. Events collected: 2000. Experiments were performed in duplicate. (**C**) Analysis of Ki-67 expression levels in T87G at increasing confluences. Ki-67 positive cells are reported in red whereas Ki-67 negative cells in blue. Events collected: 2000. (**D**) Isotypic IgG control.

# Supplementary Figure 4

| 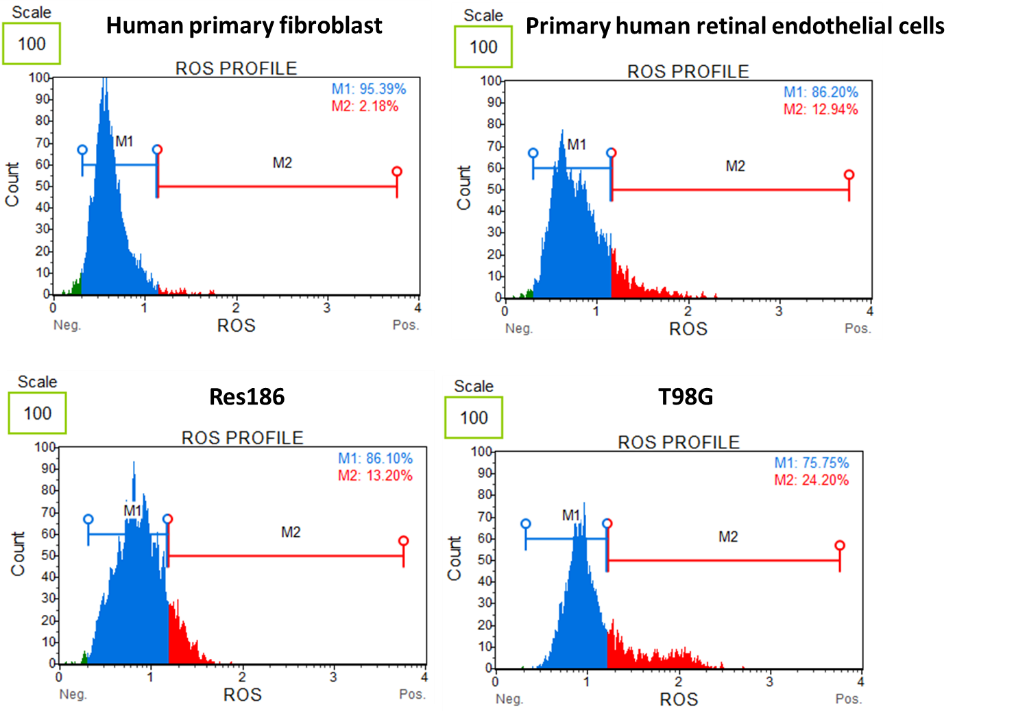 |
| --- |

**Figure S4.** Cytofluorimetric analysis of intracellular ROS levels in human primary fibroblasts and retinal endothelial cells (normal cells) as well as in two glioma cell lines, i.e., Res186 (Grade I) and T98G (Grade IV). M1 gate: ROS-; M2 gate: ROS+. Events collected: 2000 cells.

# Supplementary Figure 5

| 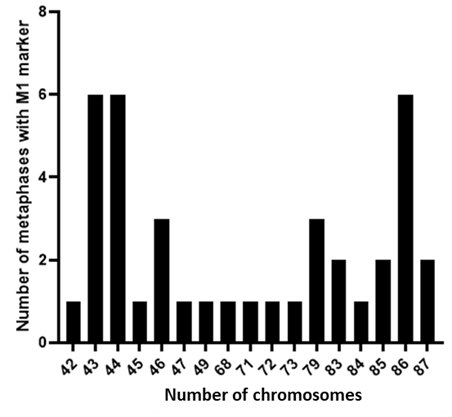 |
| --- |

**Figure S5.** Distribution of the marker M1 in ClK metaphases. On X-axis are reported the number of chromosomes whereas in Y-Axis the number of metaphases showing the M1 marker. Three significant peaks were observed in metaphases with 43, 44 and 86 chromosomes.

# Supplementary Table 4

| N° of chromosomes | N° of metaphases |
| --- | --- |
| 42 | 1 |
| 43 | 11 |
| 44 | 31 |
| 45 | 9 |
| 46 | 7 |
| 47 | 2 |
| 48 | 1 |
| 49 | 1 |
| 63 | 1 |
| 66 | 1 |
| 68 | 1 |
| 71 | 1 |
| 72 | 1 |
| 73 | 1 |
| 75 | 1 |
| 78 | 1 |
| 79 | 4 |
| 82 | 1 |
| 83 | 2 |
| 84 | 3 |
| 85 | 4 |
| 86 | 9 |
| 87 | 3 |
| 88 | 1 |
| 89 | 1 |
| 90 | 1 |
| Tot. | **100** |

**Table S4.** Numerical data regarding the distribution of chromosome numbers. The two modal values, 44 and 86 respectively, are highlighted in dark grey.

# Supplementary Figure 6

| 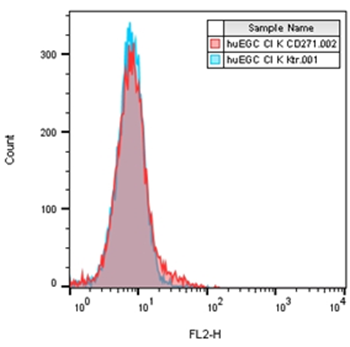 |
| --- |

# Figure S6. Analysis of CD271 expression levels in the ClK clone by flow cytometry. Light blue curve indicated unstained cells whereas red one fluorescence signals detected from cells stained with PE-labelled CD271 antibody.

# Supplementary Figure 7

| 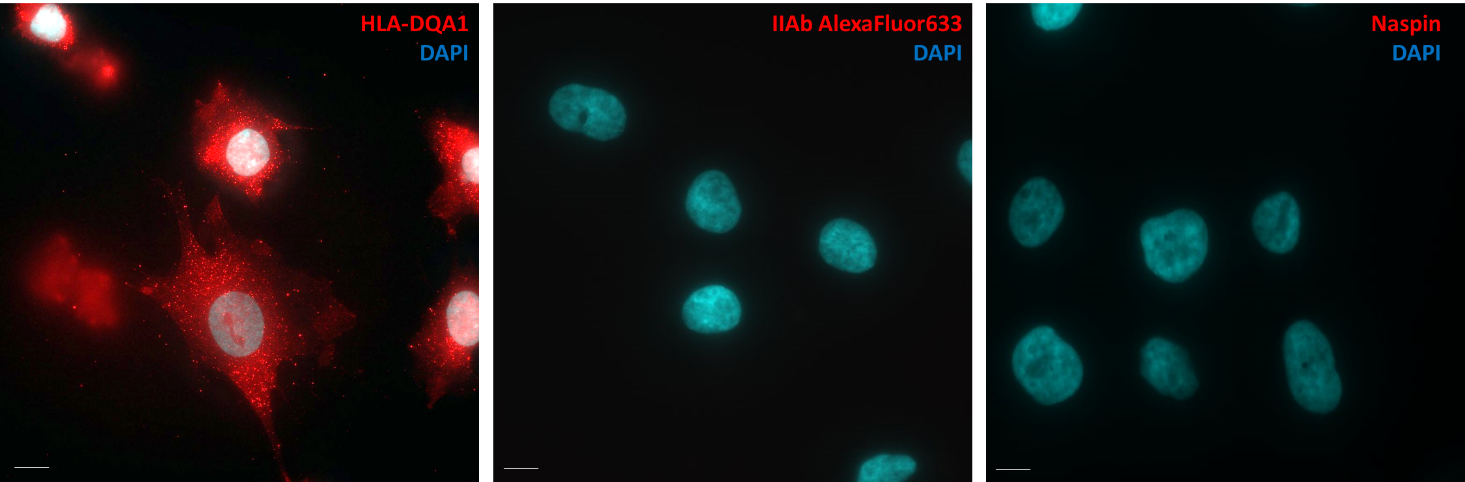 |
| --- |

**Figure S7.** Immunofluorescence of the HLA-DQA1 molecule. The analysis was performed using specific anti-HLA-DQA1 IgG antibody conjugated with AlexaFluor633. Nuclei were stained with DAPI. Scale bars (= 10 μm) are reported. Negative controls were performed using the secondary antibody alone and anti-naspin IgG antibody.

# Supplementary Table 5

| **HLA-A*** | **HLA-B*** | **HLA-C*** | **HLA-DRB1*** | **HLA-DQA1*** | **HLA-DQB1*** |
| --- | --- | --- | --- | --- | --- |
| 01:01 | 07:02 | 07:01 | 13:01 | 01:02 | 06:02 |
| 30:01 | 08:01 | 12:03 | 15:01 | 01:03 | 06:03 |

**Table S5.** HLA genotyping of ClK clone. Results were collected through PRC-SSO and subsequent validation with PCR-SSP.

# Supplementary Figure 8

| 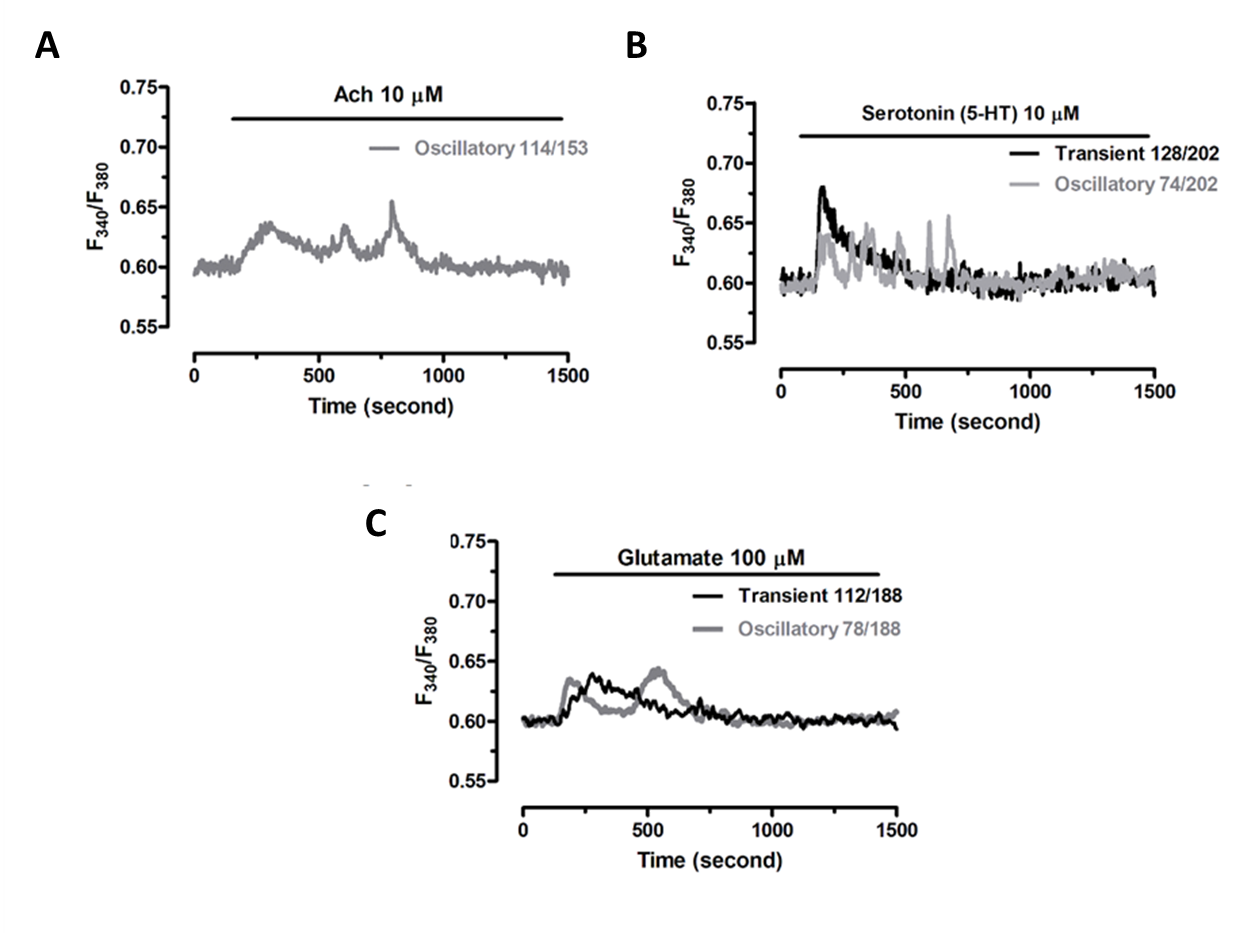 |
| --- |

**Figure S8.** Acetylcholine (Ach), serotonin (5-HT) and glutamate induce increase in [Ca^2+^]_’_ in ClK clone. (**A**) Ach (10 µM) triggered oscillatory Ca^2+^ signals in ClK cells. (**B**) The Ca^2+^ response to 5-HT (10 µM) was characterized by both transient (black line) and oscillatory (grey line) patterns. (**C**) Glutamate (100 µM) induces both transient (black line) or oscillatory (grey line) increase in [Ca^2+^].
